# Current applications of capillary electrophoresis‐mass spectrometry for the analysis of biologically important analytes in urine (2017 to mid‐2021): A review

**DOI:** 10.1002/jssc.202100621

**Published:** 2021-10-07

**Authors:** Hrušková Helena, Voráčová Ivona, Řemínek Roman, Foret František

**Affiliations:** ^1^ Institute of Analytical Chemistry Czech Academy of Sciences Brno Czech Republic; ^2^ Faculty of Science Masaryk University Brno Czech Republic

**Keywords:** capillary electrophoresis, drugs, mass spectrometry, metabolome, urine analysis

## Abstract

Capillary electrophoresis coupled online with mass detection is a modern tool for analyzing wide ranges of compounds in complex samples, including urine. Capillary electrophoresis with mass spectrometry allows the separation and identification of various analytes spanning from small ions to high molecular weight protein complexes. Similarly to the much more common liquid chromatography‐mass spectrometry techniques, the capillary electrophoresis separation reduces the complexity of the mixture of analytes entering the mass spectrometer resulting in reduced ion suppression and a more straightforward interpretation of the mass spectrometry data. This review summarizes capillary electrophoresis with mass spectrometry studies published between the years 2017 and 2021, aiming at the determination of various compounds excreted in urine. The properties of the urine, including its diagnostical and analytical features and chemical composition, are also discussed including general protocols for the urine sample preparation. The mechanism of the electrophoretic separation and the instrumentation for capillary electrophoresis with mass spectrometry coupling is also included. This review shows the potential of the capillary electrophoresis with mass spectrometry technique for the analyses of different kinds of analytes in a complex biological matrix. The discussed applications are divided into two main groups (capillary electrophoresis with mass spectrometry for the determination of drugs and drugs of abuse in urine and capillary electrophoresis with mass spectrometry for the studies of urinary metabolome).

**Article Related Abbreviations**
CC: cholangiocarcinoma
CITP: capillary isotachophoresis
CKD: chronic kidney disease
CVD: cardiovascular diseases
eGFR: estimated glomerular filtration rate
GAG: glycosaminoglycans
hCG: human chorionic gonadotropin
IBD: inflammatory bowel disease
IBS: irritable bowel syndrome
LVDD: left ventricular diastolic dysfunction
MISPE: molecularly imprinted solid‐phase extraction
pIgR: polymeric immunoglobulin receptor
PSA: prostate‐specific antigen
PSC: primary sclerosing cholangitis
RCAD: renal cyst and diabetes syndrome
SVM: support vector machines
TMAO: trimethylamine‐*N*‐oxide

## 1 INTRODUCTION

Urine is a favorable analytical biofluid especially due to its noninvasive collection [1, 2, 3]. It is an important source of biomarkers of various diseases and reflects the state of the organism. The study of these biomarkers can provide much vital information to understand how the body works [4]. Because the demands on the quality of medical treatment and cognition of organisms are continuously increasing, deeper insights into the disease's origin, mechanism of progression, or effect of the treatments are required. However, urine contains many substances that differ in molecular mass, polarity, concentration, and other properties; therefore, a suitable analytical tool needs to be applied to obtain quality analytical data [5].

The CE‐MS combines the separation ability of CE with MS identification of analytes. CE provides a high‐resolution and efficient separation of charged species [1]. Electrophoretic separation enables fast analyzes in simple aqueous media (does not require a high amount of organic solvents as LC‐MS) with little or no sample preparation. Coupling of the CE with MS results in a suitable tool for the detection of various species, positive, negative and neutral, in urine samples providing excellent sensitivity, selectivity, and identification of analytes [1, 3]. The following text summarizes knowledge and progresses in recently published studies dealing with CE‐MS analyses of compounds in urine.

## 2 URINE

**FIGURE 1:**
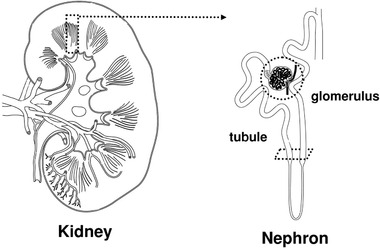
Kidney and its structural and functional components

An excretory system ensures the removal of metabolism products from mammal organisms. The kidneys, which extract waste products from the blood, play a key role, and urine is the final product of this system [5]. The human kidney contains ∼1,000,000 functional units or nephrons. These functional units consist of two parts (Figure 1). The first part is the glomerulus, which produces primitive urine by filtrating blood plasma. The second part is renal tubules that reabsorb 99% of primitive urine. The rest (final urine) is excreted via the ureter by the bladder [6, 7]. Depending on the content of individual substances, urine is colored from light to dark yellow by the pigment urobilin [5]. The average volume of urine excreted per day is approximately 1.5–2.0 L [6].

From an analytical point of view, urine is relatively easy to work with. Compared to the blood serum or plasma, which degrades proteolytically, it is a relatively stable, sterile fluid. It is available in large volumes in a short time period [8, 9], and its collection is noninvasive [5].

A wide range of analytical methods has already been used to analyze this waste fluid, providing information that allows us to gain at least partial insights into the complex metabolic processes in the organism. These include, for example, high‐resolution nuclear magnetic resonance [10], MS [11], Raman spectroscopy [12], infrared spectroscopy [13], HPLC [14], GC [15], and many other methods or their combination [8, 16].

### 2.1 Chemical composition of urine

Urine is a complex mixture of substances [5]. It consists of water (91–96%), the organic substances such as urea, ammonia, and creatinine are largely present. Other substances contained in urine are inorganic salts, organic acids, proteins, and peptides [5, 6]. It also contains trace amounts of enzymes, carbohydrates, hormones, organic acids, pigments, and inorganic ions (sodium, potassium, magnesium, calcium, ammonium, sulfate, phosphate, carbonate, and chloride) [17, 18]. The total concentration of individual elements in urine determined by Putnam [18] was 6.87 g/L carbon, 8.12 g/L nitrogen, 8.25 g/L oxygen, and 1.51 g/L hydrogen. Urine pH is normally in the neutral range (between 5.5 and 7.0). The pH value of urine is mainly affected by the composition of the diet. For example, an increased intake of alcohol, meat, or milk reduces the pH. Conversely, increased urinary potassium and organic acid intake increase urine pH [17, 19]. Overall properties and composition of urine are given in Table 1.

**TABLE 1:** The properties of urine and the contents of main urine components [26]

Property and composition	Molar mass (g/mol)	Normal range in humans (reference age in years)	Molarity (mmol/1.5 L)
Volume		0.8–2 L	
pH		4.5–8.0	
Specific gravity (SG)		1.002–1.030 g/ml (all)	
Osmolality		150–1150 mOsm/kg (>1)	
Urea (CH_4_N_2_O)	60.06	10–35 g/d (all)	249.750
Uric Acid (C_5_H_4_N_4_O_3_)	168.11	<750 mg/d (>16)	1.487
Creatinine (C_4_H_7_N_3_O)	113.12	Males: 955–2936 mg/d	7.791
		Females: 601–1689 mg/d (18–83)	
Citrate (C_6_H_5_O_7_^3−^)	192.12	221–1191 mg/d (20–40)	2.450
Sodium (Na^+^)	22.99	41–227 mmol/d (all)	92.625
Potassium (K^+^)	39.10	17–77 mmol/d (all)	31.333
Ammonium (NH_4_^+^)	18.05	15–56 mmol/d (18–77)	23.667
Calcium (Ca^2+^)	40.08	Males:<250 mg/d	1.663
		Females:<200 mg/d (18–77)	
Magnesium (Mg^2+^)	24.31	51–269 mg/d (18–83)	4.389
Chloride (Cl^−^)	35.45	40–224 mmol/d (all)	88.000
Oxalate (C_2_O_4_^2−^)	88.02	0.11–0.46 mmol/d (all)	0.277
Sulfate (SO_4_^2−^)	96.06	7–47 mmol/d (all)	18.000
Phosphate (PO_4_^2−^)	94.97	20–50 mmol/d (>18)	23.33

One of the most abundant compounds in urine is urea [20]. This metabolite is created by the body to remove waste nitrogen. The process of urea synthesis occurs in the liver. Subsequently, this compound is transferred via blood to the kidneys where it is excreted in the urine. Daily excretion is very variable [21]. Another substance present in higher concentrations in urine is creatinine. Since its amount in the muscles is relatively constant, it can be used to determine creatinine clearance. Creatinine clearance can be described as the volume of blood plasma cleared of creatinine per time. This is related to the rate of glomerular filtration, which is an important variable for assessing proper kidney function. Low creatinine clearance levels can be caused by acute kidney injury [22, 23, 24]. Increased levels indicate kidney disease or impaired kidney function. Furthermore, creatinine is also used for the normalization of analyte quantity in urine samples. The levels of analytes in urine depend on many factors (e.g., rate of urine production). Creatinine normalization procedure allows a noninvasive and fast elimination of this variation. The concentration of analyte is simply divided by the concentration of creatinine in the same urine sample. However, this method is not appropriate for all patients, because cystic fibrosis causes irregular creatinine excretion [25].

Urine is also a source of proteins and peptides. The number of identified proteins or peptides is still increasing [6]. Normal excretion of protein is less than 150 mg/L per day, and 70% of urinary protein content is created in kidneys; the rest comes from plasma via blood filtration [26]. The most abundant proteins in urine are serum albumin and uromodulin [27, 28].

Besides the traditional analyses performed in the doctor's office (e.g., presence of protein or sugar), many urine components can also serve as potential disease markers. While a single biomarker can provide some information about the state of the disease, its specificity and sensitivity are limited. Using more biomarkers and generating a panel can provide more precise prediction and discrimination between groups of patients [6]. With an increasing number of biomarkers, the demands on the measurements of samples and data evaluation also increase. For extensive metabolomic studies, proper statistical processing of the obtained data is necessary [29]. Also, for data processing and classification, support vector machines are often applied. Support vector machines is a learning machine tool that can be trained to learn rules from patterns in data [30, 31].

## 3 CAPILLARY ZONE ELECTROPHORESIS–MASS SPECTROMETRY

In the CZE, charged species migrate in the capillary filled with a background electrolyte under the influence of an applied electric field [32]. The different ionic species separate into migrating zones based on differences in their electrophoretic mobilities [33]. Since the introduction of the first commercial instruments in the 1980s, CE has evolved into a common laboratory technique for the separation of ionizable compounds.

The CE‐MS consists of CE, an interface connecting both techniques, and a mass detector [34]. It was firstly introduced in 1987 using ESI and a quadrupole mass spectrometer [35]. The combination of CE with MS provides the high‐resolution separation of the sample constituents and their identification and structural characterization [9, 36]. Since the CE separation proceeds in a liquid phase and the MS operates in a vacuum, a suitable interface is required to generate gas‐phase sample ions. While ESI is the dominant ionization technique for the coupling of MS with separation technique, also other ionization techniques, for example, MALDI [37], can be applied. With regard to that ionization, techniques other than electrospray are not discussed later in the paper, and their principles are omitted.

ESI is based on the creation of charged droplets under the influence of the electrical field. In the first step, the liquid containing analytes is dispersed using high voltage. Subsequently, the solvent from droplets evaporates and their volume decreases. After electrostatic repulsion overcomes surface tension, the droplets deform, disintegrate, and produce a large amount of smaller more stable droplets. This process continues until single ions are created [38].

Besides the electrostatic dispersion and ionization of the analyzed liquid, the ESI interface for the CE has to ensure the conductive connection for the electrophoresis high voltage source. Many different approaches have been used over the past decades of CE‐MS development [34, 39].

**FIGURE 2:**
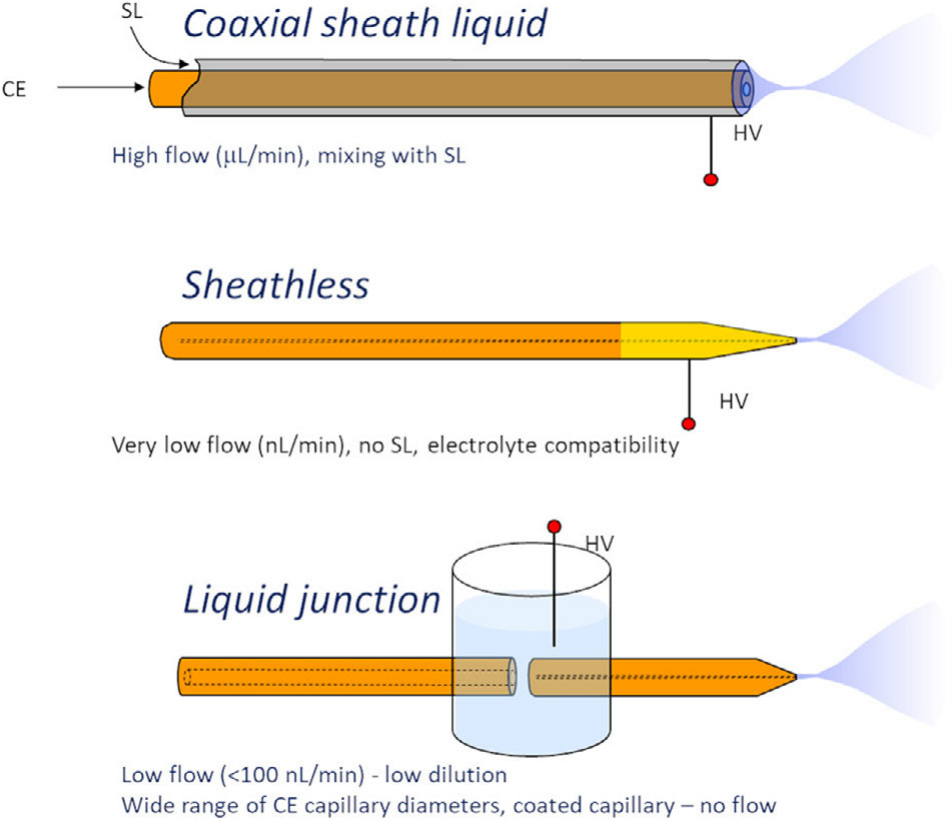
Schematic of the three most common ways to interface CE‐MS

The most common interface designs are typically divided into three categories—the sheath liquid [40], the liquid junction [41, 42, 43], and the sheathless [35, 38, 44, 45, 46] (Figure 2). The coaxial sheath liquid interface consists of a separation capillary that is placed inside of the capillary, which delivers the sheath liquid and mixes it with the sample at the capillary terminus. Both capillaries are often surrounded by a third tube delivering sheath gas improving the stability of electrospray and evaporation of the electrosprayed droplets.

The sheathless interface does not need any liquid addition for its operation; however, a flow inside the separation capillary must be established by pressure or electroosmosis to deliver the liquid into the electrospray. While the first sheathless interfaces were based on a CE capillary with a sharpened tip coated with a conductive layer for electric connection [45], currently the most often used, commercially available sheathless interfaces are based on the on‐capillary etched semipermeable junction first described by Janini et al. [47]. Here, the separation capillary is etched close to its exit to a point when the fused silica becomes permeable to small ions of the background electrolyte but still prevents liquid flow. This membrane section is then positioned inside an electrode reservoir for electric connection. In cases when a very narrow separation capillary is used (∼15 cm or less), the electrospray current may approach that of the CE separation current. In such a case, only one high voltage power supply can be used for both the CE separation and electrospray generation [48, 49].

The sheath liquid and liquid junction interfaces operate on the same principle and differ only by the mechanical arrangement. In both cases, a sheath (spray) liquid is added to the separated ions that exit the CE separation capillary. Thus, the flow inside the separation capillary is not needed, and the separation can proceed under no‐flow conditions. The sheath liquid typically contains mixed organic (methanol, isopropanol)/water solution of volatile acids or bases and serves as the electric contact for closing the CE electric circuit. The sheath liquid based interfaces typically provide lower sensitivity than the sheathless arrangement [36, 50]; however, the miniaturized versions operating in the nl/min flow ranges can approach the sensitivity of the sheathless interfaces [42, 51, 52, 53].

The liquid junction interface is an alternative to the commonly used sheath liquid interface. A sharp electrospray tip brings the efficiency and stability of the ionization to the same level as the sheathless interface (providing the flow rate and spray tip dimensions are the same). The zones exiting the separation capillary are mixed with sheath liquid at a junction through a gap between the separation capillary and electrospray needle. More technical details about different ESI interface designs can be found in recent reviews [54, 55, 56, 57].

### 3.1 Preparation and storage of urine samples for the CE‐MS measurements

Urine can be collected in a specific period (usually to observe time‐related trends), or at random times (any time of the day). After excretion of urine, proteolytic degradation by endogenous proteases is completed, so unlike blood, urine is relatively stable even at room temperature for several hours [58]. However, if urine samples need to be stored for a longer time, it is advisable to freeze them at –80℃ to eliminate residual enzymatic activity [59]. To prevent bacterial contamination, preservatives such as sodium azide and boric acid can be added to the sample. Also, the pH of urine can be adjusted. Deproteinization is commonly applied for blood samples and plasma but this step is not crucial to apply on urine samples, because of the low protein content. However, 1:5 dilution in 50% acetonitrile can be used. To eliminate materials in suspension, filtration on cellulose membrane or centrifugation can be used. Also, ultrafiltration using membranes with different cut‐offs can be applied to filter compounds from the specific molecular weight. SPME can be applied for analytes preconcentration and clean‐up. Nonpolar and low‐polar substances can be separated using liquid–liquid extraction. Also, partial evaporation can be used for the sample preconcentration. Unlike blood samples, where cellular components occur, these steps are not necessary [60].

Untreated urine or just diluted urine can be also analyzed using CE‐MS (methods called dilute and shoot). Nevertheless, such analyzes can be problematic in terms of clogging of various instrument components, and it is, therefore, advisable to adjust the sample before analysis [58].

## 4 CE‐MS FOR THE DETERMINATION OF DRUGS AND DRUGS OF ABUSE IN THE HUMAN URINE

The determination of the drugs and drugs of abuse in urine belongs to the most important CE‐MS applications. After entering the organism, these compounds undergo metabolic processes and conversions, allowing their excretion from the body [61]. One path for the elimination of the drugs from the organism leads through the liver to the excretory system. Besides the metabolic products, a small amount of unchanged drugs can be also found in urine [62]. Recent CE‐MS applications for the determination of drugs (or drugs of abuse) and their metabolites in urine are summarized in Table 2.

**TABLE 2:** The summary of articles dealing with the CE‐MS determination of drugs and drugs of abuse in urine

Analytes	Method	LOQ/LOD [ng/ml]	Reference
Acetaminophen, metabolites	CZE‐ESI‐MS/MS	LOQ 25–500	[60]
Salicyluric acid	Multisegment injection CE‐ESI‐MS	X	[61]
Oxaliplatin enantiomers	Chiral CZE‐ICP‐MS	LOQ 115, 116 (^194^Pt and ^195^Pt isotopes)	[62]
Varenicline	CZE‐MS, CZE‐MS/MS	LOQ 10 in water, 15 in urine	[63]
5‐Nitroimidazoles, metabolites	MISPE‐CZE‐MS/MS	LOQ 9.6–130.2	[59]
hCG, hCG‐based drugs	CZE‐MS	X	[64]
Azathioprine, metabolites, and co‐medicated drugs	CE‐ESI‐MS/MS	28.4–268	[65]
Drugs of abuse, metabolites	Multisegment injection CZE‐MS	X	[66]
Drugs of abuse, metabolites	Multisegment injection CZE‐MS, CZE‐MS/MS	LOD 0.4–9.6	[67]
(*R,S*)‐3,4‐Methylenedioxypyrovalerone	SPE‐CE‐MS	10	[68]

Lecoeur et al. [63] developed and validated the CE‐ESI/MS‐MS method for the determination of acetaminophen and five of its metabolites in the urine samples. The CE online coupled with the triple quadrupole mass spectrometer with the sheath liquid interface increased LOQ value 10‐ to 20‐fold (depending on the analyte) in comparison with UV detection and allowed to quantify two additional metabolites. Bare fused silica capillary (50 μm id, 80 cm length), 40 mM ammonium acetate (pH 10) as the BGE, and methanol/water (50:50 v/v) with 0.1% ammonium hydroxide as the sheath liquid were used for the experiments. The urine was diluted 20–200 times with the BGE. The method was applied to three different inclusion groups of patients with hepatic surgery/resection/re‐operation showing promising results in the differentiation between these groups. Aspirin is still one of the most frequently used drugs for pain relief and also for inhibiting platelet aggregation. Multisegment CE‐MS was used for the determination of salicyluric acid concentrations, the predominant urinary aspirin metabolite [64]. The platinum complexes with organic ligands are widely used cancerostatic drugs. CE‐MS was demonstrated for analyzes of these compounds at a very low (attomolar) concentration of oxaliplatin enantiomers. Oxaliplatin occurs as two enantiomers, and only the (*R*,*R*)‐enantiomer of oxaliplatin is considered to be the active drug. In this study, the CE‐ICP‐MS method for the separation and the determination of oxaliplatin enantiomers was developed and subsequently used for the determination of the drug in the urine samples. The BGE consisted of a 40 mM sodium borate buffer (pH 9.5) with 60 mg/mL sulfated‐β‐cyclodextrin as a chiral selector. Fused silica capillaries with id 25 μm and a total length of 64.5 cm were used. Sheath liquid was composed of 20‐fold diluted BGE without a chiral selector. For the hyphenation of the CE instrument with the ICP‐MS detector, the in‐house interface consisting of a cross‐piece, grounding electrode, sheath liquid inlet, and self‐aspirating MicroMist concentric nebulizer was used. The method was calibrated for the two platinum isotopes (^194^Pt and ^195^Pt) with similar abundance. The LOD and LOQ values were 64 and 116 ng/mL. Both enantiomers were detected in the spiked urine samples [65].

Varenicline is a drug used for the treatment of smoking addiction. Piešťanský et al. [66] developed and compared two methods for the determination of varenicline: hydrodynamically closed 2‐D capillary coupled with UV detection (capillary isotachophoresis‐CZE‐UV [CITP‐CZE‐UV]) and hydrodynamically opened CZE‐MS. Methods were subsequently tested for the determination of the drug in urine samples. Both methods were developed in terms of increasing casual CE‐UV method effectiveness. In the CITP‐CZE‐UV technique, the ITP module consisted of the polytetrafluorethylene capillary (800 μm id, 90 mm total length) and contactless conductivity detector. The CZE module differed in the capillary (id 300 μm and 160  mm total length) dimensions and was equipped with photometric detection. For the CZE‐MS method, a capillary with id 50 μm (total length 90 cm) and a triple quadrupole tandem mass spectrometer with ESI were used. The instruments were coupled using a coaxial sheath liquid interface. Both methods showed increased selectivity and specificity when compared to the CE‐UV. The CITP‐CZE‐UV provided lower LODs than CZE‐MS (1.25 ng/mL in water and 3.0 ng/mL in urine). Another advantage of the CITP‐CZE‐UV technique was a low cost per analysis. On the other hand, the CZE‐MS allowed performing experiments in a short time with simple BGE composition.

The determination of trace amounts of antimicrobial drugs in urine may require a selective extraction/concentration step. Besides the solid phase extraction techniques more selective tools may be required, including the molecularly imprinted SPE (MISPE), which was described for the extraction of 5‐nitroimidazoles with over 80% recoveries. Bare fused silica capillaries with 50 μm id and a total length of 110 cm and the voltage 28 or 25 kV were tested for both CZE and MEKC separations. The BGE consisted of 100 mM ammonium perfluorooctanoate buffer (pH 9) for MEKC mode and 1 M formic acid (pH 1.8) for the CZE mode. Sheath liquid for the CZE‐MS measurements consisted of propane‐2‐ol:water:acetic acid (60:38.8:0.2 v/v/v), the analytes were detected using ESI voltage –4.9 kV. Since the CZE provided higher selectivity, it was used in the final optimization of the MISPE‐CZE‐MS/MS method. The developed method provided LODs from 9.6 to 130.2 μg/L allowing the determination of 11 5‐nitroimidazoles in the urine samples [62].

Human chorionic gonadotropin (hCG) is a glycoprotein hormone specific to human pregnancy composed of 217 amino acids. Its potential glycosylation can lead to a high number of isoforms. Several electrophoretic modes, including the CE‐MS with triple quadrupole mass spectrometer, were used for the characterization of the different isoforms of the hCG and two hCG based drugs at the intact level from urine. This study avoided the conventional bottom‐up approach, where some information about the analytes can be lost. An uncoated fused silica capillary with id 50 μm, total length 60 cm, and effective length 51.5 cm was used for the CE. The sheath liquid contained water/methanol/formic acid (50:50:0.1 v/v/v). The solution of 800 mM formic acid, 800 mM acetic acid, and 20% MeOH (pH 2.2) served as the BGE. The electrophoretic methods introduced in this article proved the presence of the isoforms of the two hCG‐based drugs, but the identification of the isoforms was not achieved due to the limited resolution of the triple quadrupole mass spectrometer [67].

For the treatment of inflammatory bowel disease (IBD), thiopurines are commonly used. Maráková et al. [68] developed a CE‐ESI‐MS/MS method for the separation and determination of the most important thiopurine drug azathioprine and its metabolites (6‐thioguanine, 6‐mercaptopurine, and 6‐methylmercaptopurine) and co‐medicated drugs (mesalazine, prednisone, and allopurinol) in human urine. CE with uncoated fused silica capillary with 50 μm id, 85 cm total length, +30 kV voltage, and the triple quadrupole mass spectrometer with coaxial sheath liquid interface set at +4.5 kV was used in the experiments. Note that 10 mM ammonium acetate with 5% methanol (pH 9) solution was used as the BGE, and methanol/water solution (50:50 v/v) with an addition of 5 mM ammonium acetate as the sheath liquid. The developed method was used for the analysis of urine collected from a group of Crohn's disease patients treated with azathioprine. It allowed determining the levels of the drug, its metabolites, and other co‐medicated drugs with limits of detections in the range of 0.0284 – 0.268 μg/mL.

**FIGURE 3:**
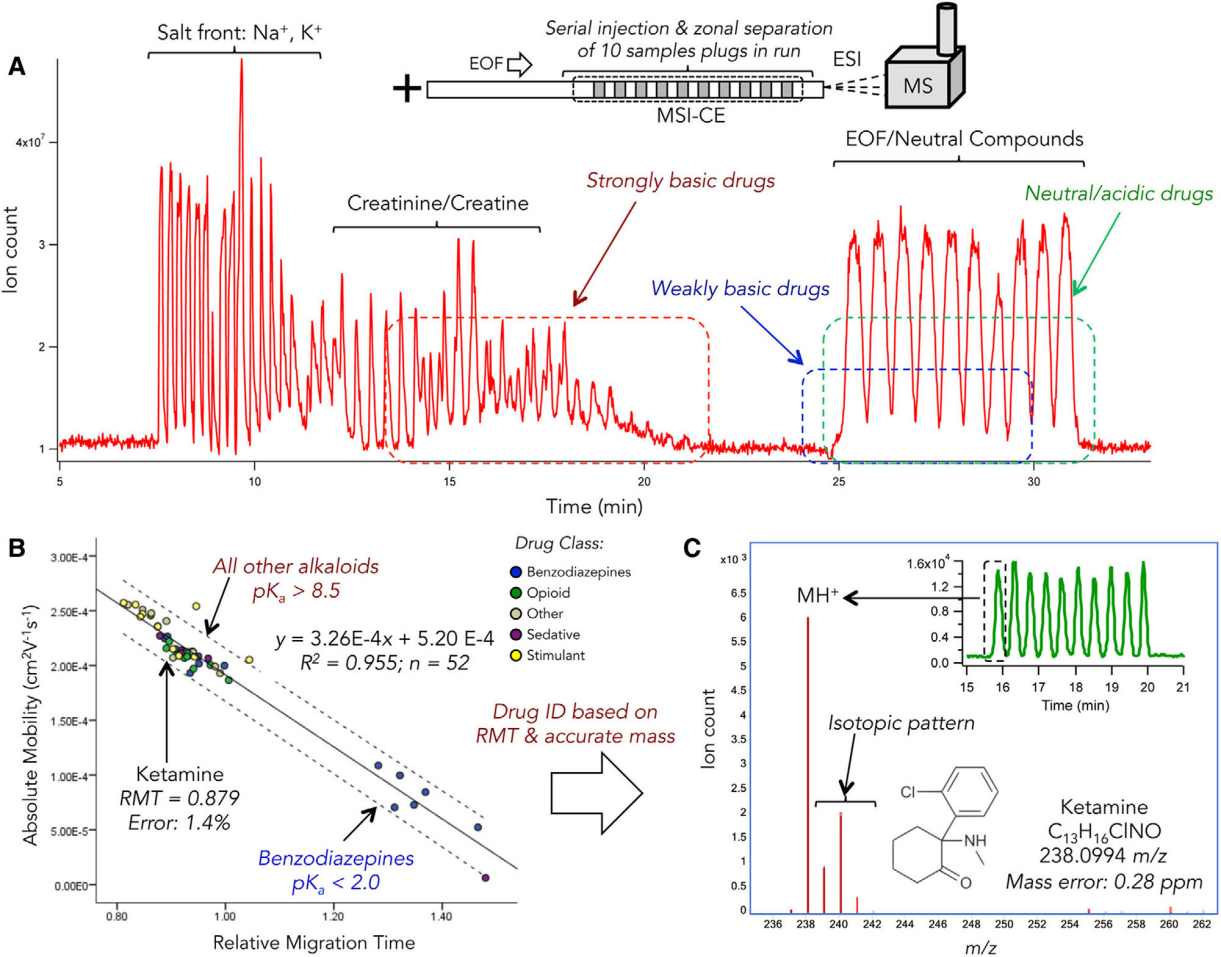
(A) Multiplexed separations for high throughput and nontargeted screening of a broad spectrum of DoA and their metabolites when using MSI–CE–MS under acidic conditions (pH 1.8) with full‐scan data acquisition and positive ion mode detection, where 10 discrete sample plugs are analyzed within a single run. A TIE depicts the resolution of major electrolytes/solutes in a synthetic urine matrix from two distinct classes of DoA, namely a large fraction of fully ionized (e.g., opioids) and weakly basic compounds (e.g., certain benzodiazepine analogs) from neutral/acidic drugs (e.g., barbiturates) that comigrate with the EOF. (B) A linear regression model with a 95% confidence interval (dashed line) that demonstrates accurate prediction of the relative migration time (RMT) of a panel of DoA (*n* = 52) based on their characteristic absolute electrophoretic mobility (p*K*_a_ and molecular volume) that facilitates identification of ketamine (*m*/*z* 238.0994; RMT = 0.879) when coupled to (C) high‐resolution MS for determination of the most likely molecular formula for its protonated molecule (MH^+^) with low mass error (<1 ppm). Note that electrolytes in synthetic urine were detected as their salt formate clusters for sodium and potassium that migrate prior to DoA and their metabolites. Adopted from ref. [70]. Open access

When improved throughput is required, a multisegment CE‐MS offers the possibility of serial injections within a single run. In the two studies [69, 70], rapid and efficient determination of several drugs of abuse and their metabolites in the urine samples was achieved using this technique. The process of drug screening is shown in Figure 3. Developed methods offer low cost and fast analyses with low consumption of solvents compared to the conventionally used LC‐MS and GC‐MS.

The in‐line SPE‐CE‐MS method was developed for the enantiodetermination of synthetic cathinone (*R,S*)‐3,4‐methylenedioxypyrovalerone in urine. After ingestion, the residue of this drug can be found in the urine at low concentration levels. The pre‐extraction step to CE‐MS enantioseparation enabled the determination of these compounds with LODs of approximately 10 ng/mL for both enantiomers [71].

## 5 CE‐MS AS A TOOL FOR METABOLOMIC STUDIES

Metabolomic studies describe variations of metabolic levels that can be associated with diseases, treatments, or the state of the organism [72].

CE‐MS is used far less than HPLC for these applications, mainly because of reproducibility concerns. Recently, some effort has been put into assessing the reliability of the CE‐MS for the determination of urinary metabolite concentrations [73]. Additionally, testing the reliability of uncharacterized peaks in the urinary untargeted metabolomics was also performed [74] including relative migration‐time reproducibility and identification capabilities in an interlaboratory trial [75]. For annotation of metabolites with a higher confidence level, an internal library platform of 226 standards together with their in‐source fragment ions, adducts, and relative migration times was developed [76].

Several articles describing the connection between urine composition and diseases using the data from the CE‐MS were recently reported. Valuable data were obtained for phenylketonuria [77], rheumatoid arthritis [78], systematic lupus erythematosus [79, 80], chronic obstructive pulmonary disease [81], chronic graft‐versus‐host disease [82], liver fibrosis [83], and periprosthetic joint infection [84]. Besides the study of various diseases, this technique allowed the generation of the biomarker classifier of mice aging applicable to humans [85], the prediction of 1‐year mortality after discharge from the intensive care [86], and rapid screening of biomarkers indicating recent smoke exposure [87].

Other articles describing CE‐MS for metabolomic studies can be divided into three groups: the articles dealing with the diseases of the excretory system, cardiovascular diseases, and diseases connected with the digestive tract. Papers dealing with CE‐MS used for the metabolomic studies are summarized in Table 3.

**TABLE 3:** Articles dealing with the determination of urine metabolome

Object of studies	Analytes in urine	Number of analytes or (potential) urinary biomarkers	Reference
Reliability of urinary metabolomic CE‐MS profiling	Metabolites	123	[70]
Reliability of uncharacterized peaks	Untargeted analytes	74 Peaks	[71]
CE‐MS reproducibility, identification capability	Cationic metabolites	21 Compounds	[72]
CE‐MS search platform for metabolites annotation	Metabolites	226	[73]
Phenylketonuria	Phenylalanine, metabolites	12 Biomarkers	[74]
Rheumatoid arthritis	2‐Quinolinecarboxylic, various metabolites	6 Biomarkers	[75]
Lupus erythematosus	Peptides	65 Biomarkers	[76]
Lupus erythematosus	Peptides	273 (CKD biomarker panel), 172 (LN biomarker panel)	[77]
Chronic obstructive pulmonary disease, alpha‐1 antitrypsin deficiency	Peptides	66 Biomarkers	[78]
Chronic graft‐versus‐host disease	Peptides	14 Biomarkers	[79]
Liver fibrosis	Peptides	50 (Biomarker panel)	[80]
Periprosthetic joint infection	Peptides	137,83,70 (Biomarker panels)	[81]
Ageing	Peptides	49 Biomarkers	[82]
Mortality after discharge from intensive care	Peptides	128 (Biomarker panel), 19 individual urinary peptides	[83]
Screening method for monitoring of smoke exposure	1‐Hydroxypyrene glucuronide	1	[84]
Albuminuria and spironolactone treatment	Peptides	273 Biomarkers	[88]
Prediction of mortality and cardiovascular disease	Peptides	273 Biomarkers	[89]
Degree of fibrosis	Peptides	273 (Biomarker panel), 5 individual peptides	[90]
Progressive eGFR loss	Peptides	296	[91]
Renal processing of peptides in CKD patients	Peptides	6278 in total, 1580 sequenced	[92]
Renal processing of peptides	Peptides	3955 in total, 1461 sequenced	[93]
Comparison of amniotic fluid and fetal urine	Peptides	67 Biomarkers	[94]
Improvement of eGFR	Peptides	141 Biomarkers	[95]
Progression of end‐stage renal disease	Peptides	20 Biomarkers	[96]
Chronic active antibody‐mediated rejection in kidney transplantation children	Peptides	79 Biomarkers	[97]
Differentiation of the CKD types	Peptides	2305 Biomarkers	[98]
Renal cyst and diabetes syndrome	Peptides	146 biomarker	[99]
Inflammation caused by ureteral stents	PGE2, PDG2	2	[100]
The unspecified disease of the excretory system	N‐Glycosaminoglycans	10 GAGs, several GAGs compositions	[101]
Influence of gut microbiota on uremic Solute	Uremic metabolites (amino acids, uremic Toxins, short‐chain fatty acids, etc.)	11 Microbiota‐derived uremic solutes, seven uremic toxins, Short‐chain fatty acids and urea, 19 amino acids	[102]
CKD in dogs	Peptides	133 Biomarkers	[103]
IBD	Urinary metabolites	132 Metabolites	[107]
IBD	Biogenic amines	12	[105]
Urinary serotonin	Serotonin	1	[108]
IBD	Proteinogenic amino acids	20	[109]
Aminoacids	Aminoacids, Metabolites	11 Aminoacids, 122 urinary metabolites	[110]
IBS	Metabolites	10 Biomarkers	[111]
Heart failure	Peptides	85 Biomarkers	[113]
Diastolic left ventricular dysfunction	Peptides	85 Biomarkers	[114]
β‐Blockade, heart rate	Peptides	1152 Biomarkers	[115]
Cardio‐renal syndrome	pIgR peptides	23	[116]
Cardiovascular biomarkers	Trimethylamine‐N‐Oxide (TMAO), l‐carnitine, creatinine	3	[117]
Hypertense in pregnancy	Peptides	123 Biomarkers	[118]
General cancer urinary pattern	Peptides	193 Biomarkers	[120]
Cholangiocarcinoma	Peptides	30	[124]
Cholangiocarcinoma	Peptides	2092 (Mean value in the validation set)	[123]
Colorectal cancer	Metabolites	154	[125]
Prostate cancer	Peptides	19 Biomarkers	[126]
Prostate cancer	PSA forms, N‐glycans	6 PSA forms, 77 N‐glycans	[127]
Prostate cancer	Glycopeptides	67	[128]
Prostate and bladder cancer	Metabolites under 5kDa	468 for untargeted, 6 targeted	[129]
Prostate and bladder cancer	N‐Glycans compositions	145	[130]

### 5.1 CE‐MS for the determination of diseases of the excretory system

Chronic kidney disease (CKD) is a global problem and affects up to 10% of the population in the developed countries. CKD results from chronic anomalies in the structure of the kidney, leading to its damage. There is currently no effective treatment for CKD due to the limited early stage diagnosis possibilities. The current diagnosis is based on the determination of the estimated glomerular filtration rate (eGFR). It is also possible to evaluate the urine albumin/creatine ratio [88]. However, these methods require at least a half loss of kidney function for the diagnosis of CKD. An alternative way is to perform a kidney biopsy, which is not always suitable—it is an invasive method, depends on the exact place of sampling, and cannot be repeated in a short period [89]. Therefore, in 2010 a new CKD biomarker panel consisting of 273 urinary peptides and proteins ranging from 800 to 17,000 Da was generated. The following text describes the CE‐MS conditions and preparation of samples used for the development of CKD273. The articles mentioned below adapt the same separation conditions.

The original CE‐MS method was performed with the commercial CE system online coupled with a MS instrument. Samples were treated with 2 M urea, 10 mM NH_4_OH, and 0.02% SDS. Proteins of higher molecular mass and salts were removed using ultrafiltration devices and desalting columns equilibrated with NH_4_OH. The samples were lyophilized and stored at 4°C. Before analysis, the samples were resuspended in water [90]. After the CE‐MS experiments, a large amount of the CE‐MS data need to be correctly evaluated. Algorithms including support vector machines, which can combine several biomarkers and other statistical methods are often used for these purposes [88].

The applications of the CKD273 classifier were described for its ability to predict the effects of spironolactone treatment of albuminuria in patients with type II diabetes and hypertension [91], or as a tool for the prediction of mortality and cardiovascular disease in patients with type II diabetes and microalbuminuria [92]. Magalhães et al. [93] studied the correlation between this urinary proteomic classifier and individual urinary peptides with the degree of fibrosis. A significant and positive correlation confirmed that CKD273 can be used for the prediction of the degree of fibrosis. Moreover, seven individual peptides (especially collagen fragments) were negatively associated with the degree of fibrosis. The CKD273 was also used to improve the prediction of the progressive estimated glomerular filtration rate loss, which is connected with CKD. The CKD273 subclassifies were introduced for the classification of the different stages of the CKD. The study showed that it is possible to predict the rapid loss of eGFR in patients in the early stages of CKD, individuals without CKD and high‐risk individuals without CKD [94].

The renal peptide handling in patients with CKD was also studied by measuring the abundance of peptides in urine, plasma, and spent hemodialysate. The peptide composition from these three groups of samples was correlated. The study found no correlation between peptide abundance in urine and plasma. The correlation between spent hemodialysate and plasma was moderately strong. The correlation between spent hemodialysate and urine was strong but lower than expected. The possible reason for this is that kidney processing significantly changes the composition of peptide abundance. Another observation of this study was that the most abundant peptide in the urine of CKD patients was albumin. On the contrary, most of the peptides in healthy controls urine were collagen fragments [95]. The differences between urine and plasma peptides in healthy subjects were studied using CE‐MS and CE‐ and LC‐MS/MS analyses [90]. The results of this study are in agreement with the theory that the proximal tubule reabsorbs the majority of plasma peptides. Thus, the overlap between plasma and urine peptides is low. For that reason, the majority of plasma peptides (expect collagen fragments) were not determined in the urine (resulted only in 90 overlapping peptides) [96]. In another study, Fédou et al. [97] compared the composition of amniotic fluid and fetal urine peptidome. The compositions largely correlated; thus, both amniotic fluid and fetal urine can be potentially used as biomarkers connected to developmental kidney disease.

The new potential biomarkers or classifiers connected with the diseases of the excretory system were introduced in several articles, including an improvement of the eGFR in CKD patients [98], possible progression of the end‐stage renal disease in patients with the genetic disorder [99], or for identification of chronic active antibody‐mediated rejection in pediatric kidney transplantation children [100].

Siwy et al. [101], in their study, introduced the distinguishing of different types of CKD triggered by other morbidities, namely primary focal segmental glomerulosclerosis, IgA nephropathy, minimal‐change disease, membranous nephropathy, diabetic nephropathy and hypertensive nephrosclerosis, lupus nephritis, and vasculitis‐induced kidney disease. For this purpose, identifying potential biomarkers from urine using the CE‐MS data from the Human Urinary Proteome database was performed, and the results were verified by validation. For each type of CKD, several potential biomarkers were determined and combined into classifiers allowing the discrimination of each kind of CKD with very good or excellent accuracy. The study also provided outcomes that could be possibly used for a closer description of the pathophysiology of the diseases.

**FIGURE 4:**
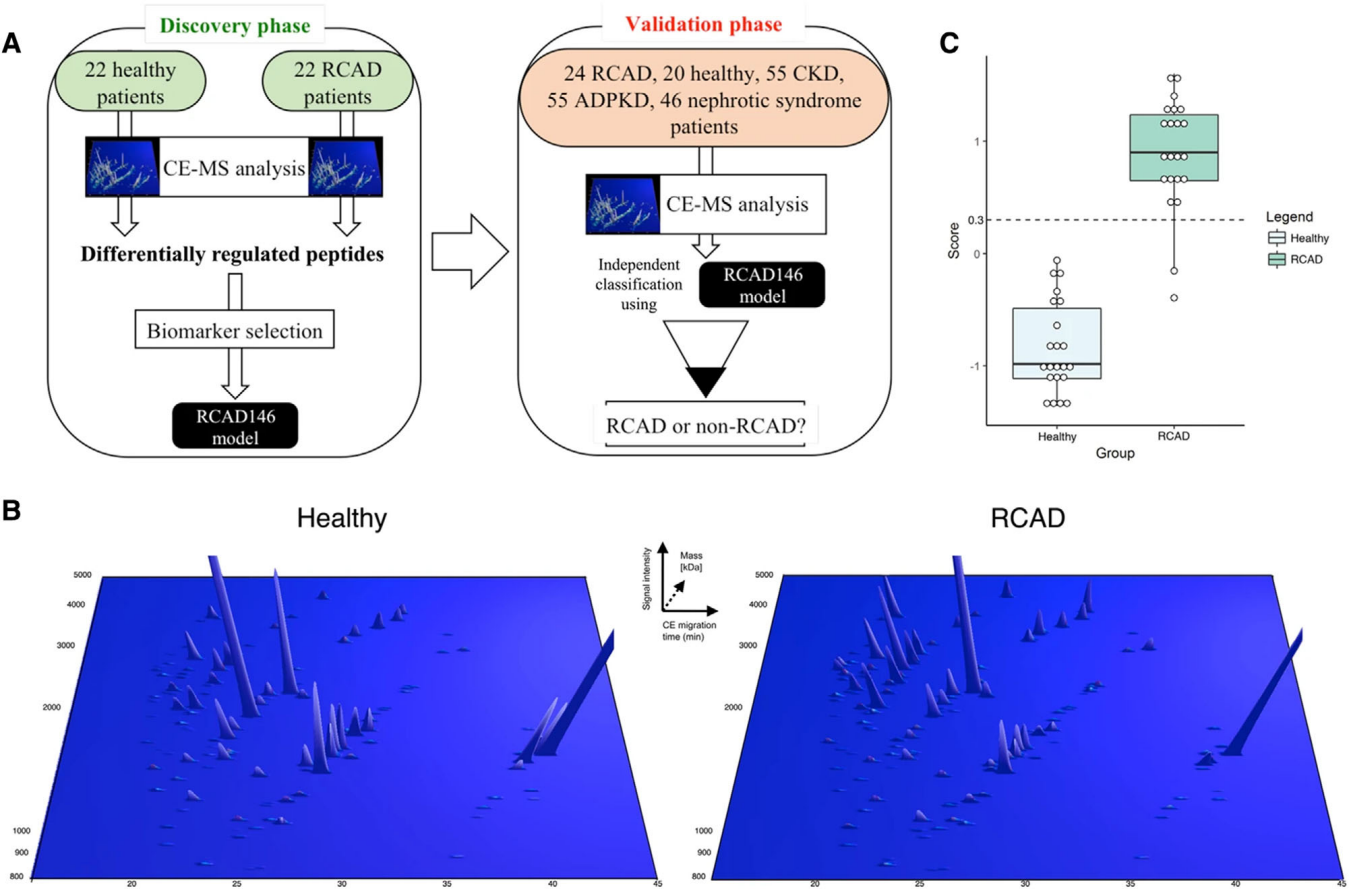
Study design and urinary CE‐MS analysis of patients with renal cyst and diabetes syndrome (RCAD). (A) The analysis was performed in two phases: a discovery phase, where the urinary proteome of 44 pediatric patients (22 healthy, 22 RCAD) was analyzed, leading to the identification of 146 sequenced urinary peptides that were modeled in a support vector machines (SVM) classifier called RCAD146. In the next step, the validation phase, we studied the discriminatory ability of the panel RCAD146 panel in new RCAD patients (*n* = 24) and individuals with chronic kidney disease (CKD) or patients carrying monogenic mutations associated with different renal diseases. (B) Representation of the 146 urinary peptides significantly modified between RCAD and healthy controls. Normalized molecular mass (kDa) was plotted against normalized CE‐migration time (min). Mean signal intensity was given in three‐dimensional depiction. (C) Cross‐validation score of the RCAD146 model from the analysis of the discovery cohort along with the definition of the cut‐off 0.3 (dashed line). Adopted from reference [102]. Open access

Renal cyst and diabetes syndrome (RCAD) in children is a genetic disorder with several symptoms, including renal abnormalities and diabetes mellitus. Ricci et al. [102] introduced a classifier based on the 146 peptides allowing discrimination of RCAD patients from healthy controls and also RCAD patients from other kidney disease patients with overall 91.67% sensitivity and 94.32% specificity. Another outcome of this study is that RCAD disease can be characterized by increased urinary collagen fragments, and decreased osteopontin and uromodulin. Design and CE‐MS analysis of patient's urine samples with RCAD are shown in Figure 4.

**FIGURE 5:**
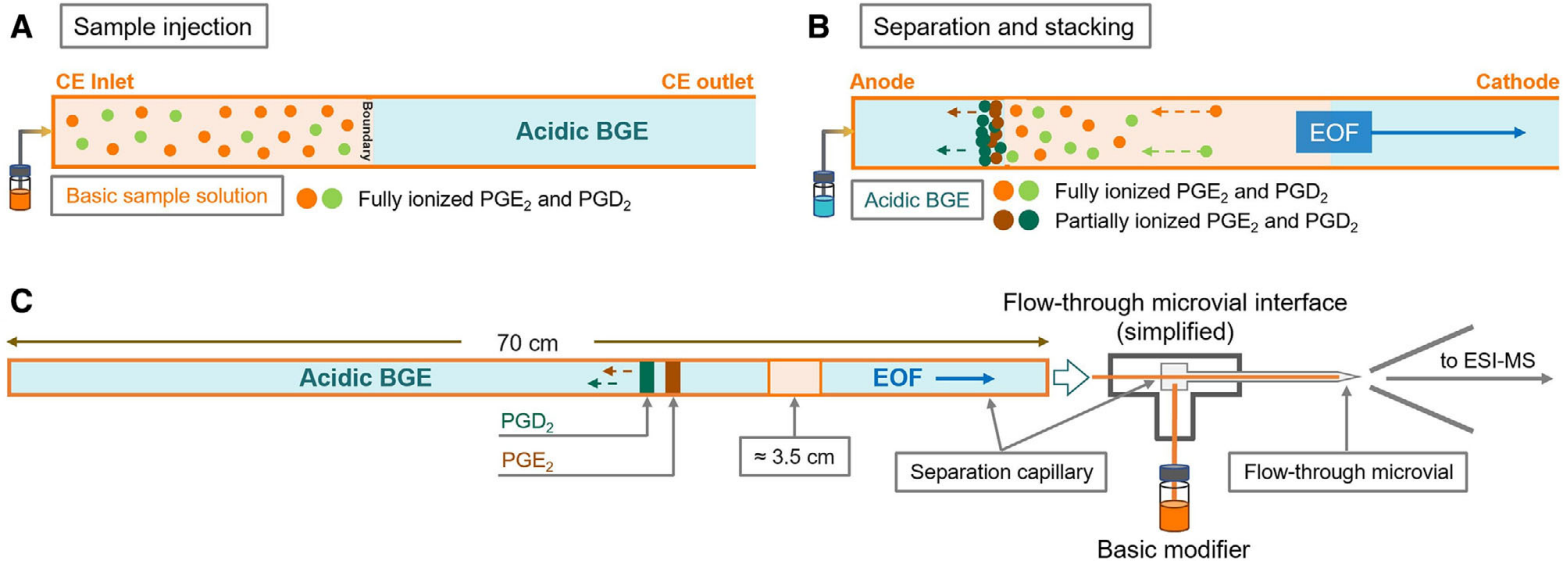
The simplified diagrams of (A) the hydrodynamic sample injection, (B) the separation and stacking of analytes under the influence of the electric field, and (C) the flow‐through microvial interface. Adopted from reference [103]. With permission from Elsevier

Ureteral stents are thin tubes implanted in the ureter, which support the urine flow from the kidneys to the ureter. Although these implants are commonly used, their placement can, in some cases, cause side effects, including inflammation. Huang et al. [103] studied the expression of cyclooxygenase‐2 and the production of its metabolite urinary prostaglandin (PGE_2_) associated with urinary inflammatory diseases in the urine samples of pigs using the nonaqueous CE‐MS method (Figure 5). The SPME was used for sample cleanup and preconcentration. The analytes were subsequently concentrated during the CE separation using online sample stacking. In the experiments, uncoated 50 μm id, 70 cm total length capillary, and voltage 28.5 kV were used for the separation and triple quadrupole mass spectrometer for the zone identification. Similar CE‐MS method was developed for the separation and determination of PGE_2_ from its geometric isomer prostaglandin PGD_2_ using BGE methanol/acetonitrile/water (8:1:1 v/v/v) containing 20 mM ammonium acetate and 0.025% acetic acid (pH 5.3). The concentration of NH_4_OH in the sample was optimized to 0.1% v/v in methanol/acetonitrile/water (9:9:2 v/v/v) solution. For the CE‐MS connection, a laboratory constructed flow‐through micro‐vial interface was developed, which offered the possibility to alkalize the acidic pH of the BGE before the MS analysis in the negative mode. This improved the signal intensity and stability. The connection between the expression of cyclooxygenase‐2 and inflammatory diseases was observed, and methodology for the quantification of PGE_2_ in the stented individuals was introduced. The scheme of the separation protocol is shown in Figure 5.

Several diseases of the excretory system (bladder disease, kidney pathogenesis, urinary tract infections) are related to the urinary glycosaminoglycans (GAG). Han et al. [104] used a negative‐ion mode nanoelectrospray ionization source for the CE‐MS with orbitrap mass spectrometer for the GAG determination using a cation‐coated capillary. The final mixing sheath liquid volume was 15 nL/min. The nanoelectrospray voltage ranged from –1.85 to –1.9 kV. The solution of 25 mM ammonium acetate in 70% methanol was used as the BGE and sheath liquid. The major components of the urinary GAGs and the structural description of the ten most abundant GAG oligosaccharides were reported.

In animal studies, the mice urine samples were analyzed to determine the influence of gut microbiota on the composition of uremic solute in CKD [105]. Pelander et al. [106] studied the peptides in dog urine to find a link to CKD. In the latter study, urine samples from 25 dogs with CKD and 25 healthy dogs were used. The study describes 133 significantly different peptides, and 35 of them were sequenced resulting in two urinary peptide biomarker models 133P and 35P, for the discrimination of healthy and CKD dogs with similar specificities (70 and 80%, respectively). The method was validated and allowed discrimination between healthy and CKD dogs in the independent group of 20 dogs.

### 5.2 CE‐MS of urine for the determination of bowel diseases

The term IBD includes several diseases associated with the gastrointestinal tract's chronic inflammation and epithelial injury. The two most important types of inflammatory bowel diseases are Crohn's disease and ulcerative colitis [107, 108]. These diseases are connected with high morbidity and can significantly affect the quality of a patient's life. While the number of IBD patients increases, the leading causes of the IBD mostly remain unknown. Therefore, the development of new analytical and diagnostical tools for the study of this disease is of high importance [109].

For example, the study of urinary metabolome of pediatric patients with Crohn's disease and ulcerative colitis using multisegment CE‐MS identified several biomarkers such as indoxyl sulfate, amino acids threonine, and serine enabling the differentiation between these diseases [110]. Maráková et al. [108] developed and validated the CE‐ESI‐MS/MS method for the association of the biogenic amines in human urine with inflammatory bowel disease. Twelve biogenic amines were separated using uncoated fused silica capillary with id 50 μm, total length of 85 cm, voltage 30 kV. A triple quadrupole mass spectrometer with sheath liquid coaxial interface and ESI was used as the detector. The solution of 50 mM formic acid (pH 2) was used as the BGE, 50% v/v methanol with 0.1% formic acid was used as the sheath liquid. Working solutions of standards and urine samples were prepared by dilution in the mixture of water with methanol. The study included the determination of biogenic amines in clinical urine samples from the patients with Crohn's disease treated with azathioprine and healthy control volunteers. The detection limits ranged between 4.47 and 144 ng/mL. In patients with inflammatory bowel disease, levels of serotonin and norepinephrine were significantly decreased while levels of histamine and spermidine were increased. Thus, these biogenic amines could be considered as the potential biomarkers of inflammatory bowel disease. Serotonin in human urine was also studied by Piešťanský et al. [111]. The group developed a highly sensitive 2‐D CITP‐CZE MS method provided the LOD approximately 34 pg/mL.

Amino acids are connected with intestinal growth and mucosal integrity; therefore, they can be potentially associated with IBD. Piešťanský et al. [112] investigated amino acid composition in the urine of patients with IBD. The fast CE‐MS/MS method for the separation of 20 amino acids was developed and tested on the clinical samples of 13 patients with Crohn's disease and a control group of 10 healthy individuals. The obtained samples were just simply diluted and filtered. Fused silica capillary with id 50 μm and total length 90 cm, positive polarity mode (+30 kV), and triple quadrupole tandem mass spectrometer with a coaxial sheath‐flow electrospray interface (capillary voltage 4 kV) was used to perform the experiments. Method for the separation of amino acids was optimized and 500 mM formic acid solution was chosen as a BGE. An optimum sheath liquid composition was methanol/water (50:50 v/v) + 5 mM ammonium acetate. The study discovers (in agreement with previously published literature) that levels of several amino acids of healthy controls and IBD patients vary. They conclude that Val, Gln, and Arg can be considered as the new potential biomarkers introduced by this study. An interesting demonstration of increasing the sensitivity of the CE‐MS method for the determination of amino acids in urine was introduced by Oedit et al. [113]. The successful connection of single‐drop microextraction with CE‐MS increased the LOD 50–250 times in comparison with conventional CE‐MS.

Another disease affecting the digestive tract is irritable bowel syndrome (IBS) characterized by abdominal pain and other symptoms. Yamamoto et al. [114] studied urine metabolome and potential biomarkers connected with this disease. For these purposes, urine samples of 42 patients were compared with the samples of 20 healthy individuals using CE‐MS. CE with uncoated fused‐silica capillary (id 50 μm, total length 110 cm) online coupled with TOF mass spectrometer or quadrupole TOF mass spectrometer using coaxial sheath liquid electrospray were used in these experiments. Separations of cations were performed in a 1 M formic acid buffer containing 15% v/v acetonitrile (pH 1.80). Anions were separated using 50 mM ammonium bicarbonate (pH 8.5). The sheath liquids consisted of 60% methanol with 0.1% formic acid for the positive mode, and 50% methanol for the negative mode, respectively. Several urinary metabolites (glycosylated hydroxylysine metabolites, amino acids, nucleotides, and/or their modified analogs and catabolites) were consistently increased in the urine of IBS patients. The study also provides partial insights into the mechanism of IBS and supports the assumption of the mucosal layer degradation and the presence of chronic low‐grade inflammation in IBS patients. After validation, metabolites introduced in this study could be used as the IBS biomarkers. In the future, the introduced method could replace uncomfortable physical examinations connected with the IBS diagnosis.

### 5.3 CE‐MS of urine for the determination of cardiovascular disease

The term cardiovascular disease (CVD) includes a wide range of disorders connected with the cardiovascular system. CVD is one of the world's most frequent causes of death, and finding new tools enabling a thorough examination of CVD is of key importance [115].

The following three articles study the potential of a novel classifier HF1 based on 85 urine peptide fragments. Campbell et al. [116] studied the urinary proteome of 829 individuals (622 with chronic or acute heart failure and 207 healthy controls). The study founds that the HF1 classifier allows discrimination between patients with heart failure, healthy controls, and coronary heart disease (but without heart failure). Results are comparable to the commonly used B‐type natriuretic peptide test. HF1 was also used by Zhang et al. [117] to investigate left ventricular diastolic dysfunction (LVDD), which can evolve into heart failure. Diastolic heart failure causes 50% of all heart failures and 30% end with death. HF1 urinary classifier was tested as a tool for discrimination between normal and mildly impaired diastolic LVDD in comparison with echocardiography in 5 years horizon. The study confirms that HF1 enables to screen LVDD in asymptomatic patients.

The association of HF1 with HF2, ACSP75, CKD273 classifiers, and individual peptides with β‐blockade treatment and heart rate in patients with heart transplantation was studied by Huang et al. [118] using CE‐MS. The study found that β‐blockade treatment but not heart rate is connected with the above‐mentioned classifiers. He et al. [119] used CE‐MS data from Human Urine Proteome Database to study the link of urinary polymeric immunoglobulin receptor (pIgR) peptides with the cardio‐renal syndrome. The study discovered the correlation between 23 pIgR peptides with eGFR and cardiovascular disease and provides insights into pIgR cleavage.

The CE‐UV‐MS/MS method for the determination of possible cardiovascular biomarkers trimethylamine‐*N*‐oxide (TMAO), l‐carnitine, and creatinine in human urine was developed and validated. The method is based on dual detection because of the high concentration of creatinine in the samples. CE with fused silica capillaries (21.5 cm effective length to the UV detector, 92 cm total length to the MS detector with id 50 μm) coupled with ESI triple quadrupole mass spectrometer (positive ionization mode +4.5 kV) was used for these purposes. The best results were achieved with 0.1 M formic acid as BGE and 70:30 methanol:water containing 0.05% v/v formic acid as sheath liquid. Limits of detection for TMAO, l‐carnitine, and creatinine were 0.76, 0.54, and 303 μmol/L, respectively. The levels of TMAO and L‐carnitine were significantly elevated in the urine of patients with cardiovascular events [120].

Another interesting application of CE‐MS is the study of hypertension during pregnancy. Hypertension is a widespread complication that can increase the probability of maternal or fetal mortality. The connection between the urinary peptidome of pregnant animals (rats) and hypertension was found, and the role of the glycoprotein uromodulin in hypertension was outlined [121].

### 5.4 CE‐MS of the urine for the diagnosis of cancer

Cancer, uncontrolled growth of cells, is one of the main causes of death worldwide. Because there are many types of cancer caused by different mechanisms and processes, the demands on the diagnosis techniques are high [122, 123]. CE‐MS has already proved its diagnostic potential for cancer studies in combination with multi‐marker patterns [124, 125].

Belczacka et al. [123] used previously published CE‐MS data to determine a general urinary marker pattern associated with solid tumors and related inflammation. A group of 2055 urinary profiles consisted of patients with different kinds of tumors (bladder, renal cell carcinoma, and more), and healthy controls were examined. In total, 193 tumor‐specific peptides (probably related to general systemic effects during cancer progression) were associated with five types of cancer.

Cholangiocarcinoma (CC) affects the bile ducts and represents 3% of all gastrointestinal cancers. The diagnosis of CC can be a difficult task regarding other biliary diseases that can also develop in patients suffering from CC. For proteomics, it is a challenging task to differentiate CC from other biliary disorders in the early stage. The following two articles deal with the diagnosis of this disease based on the data from the urinary proteome. Voigtländer et al. [126] examined bile and urine proteome analysis for the detection of CC in patients with primary sclerosing cholangitis (PSC). In this study, urine samples from a group of 87 patients with PSC, CC patients (partially with PSC), and other benign disorder patients were analyzed. The experimental results show that CE‐MS proteomic analyses of bile and urine can provide better sensitivity and specificity than existing diagnostic tools. The same group used the same CE‐MS technique also for protease mapping connected with CC using urine peptide profiles [127].

Almost 2 million new cases of colorectal cancer and nearly 1 million deaths occurred in 2018. Therefore, Udo et al. [128] studied metabolites connected to this disease in the urine of patients. Overall, 154 metabolites in 247 subjects were quantified and their differentiation ability was tested.

The next very often occurring cancer is prostate cancer. It is diagnosed to 15–20% of men during their life. However, the mortality is low because effective treatment of the slowly progressive low‐risk cancer forms is available. The noninvasive method for improving high‐risk prostate cancer differentiation from low‐risk forms was developed in the study by Frantzi et al. [129]. The CE coupled with the orbitrap MS technique was used for the determination of urinary peptides associated with high‐risk prostate cancer. A new biomarker model based on 19 significant peptides was established with 59% specificity and 90% sensitivity after validation. In the future, the test could help reduce the number of invasive biopsies to determine significant forms of prostate cancer. Another promising tool for the determination of prostate cancer is the newly developed prostate‐specific antigen (PSA) assay based on measuring urinary forms of PSA using the CE‐MS [130, 131]. CE coupled with QqTOF mass analyzer via a sheathless ESI interface was used for the PSA glycoform assay development. The assay was used for the differentiation of prostate cancer types [131]. The human urine metabolome under 5 kDa in patients with prostate and/or bladder cancer was examined by MacLennan et al. [132]. The metabolome analyses were performed using the CE with 50 μm id and 85 cm total length fused silica capillary coated with cationic polymer trimethoxysilylpropyl polyethyleneimine‐HCl in isopropanol 50% v/v. The CE was coupled with an ESI triple quadrupole mass spectrometer. The BGE and chemical modifier consisted of formic acid, methanol, and water in different ratios for different types of analytes. The experiments can be divided into targeted and untargeted analyses. Targeted measurements were correlated to creatinine concentrations and provided detection and determination of the endogenous concentration of five amino acids and sarcosine. Untargeted measurements included the determination of 468 compounds. For the discovery of significantly changed levels of analytes, the principal component analyses with six components were applied. Nine *m*/*z* values were significantly increased or decreased compared to the control healthy group. Another application of the CE‐MS was the identification of N‐glycans and sulfated N‐glycans composition present in the urinary exosomes (small membrane vesicles), which can be considered as the potential candidates for identifying bladder or prostate cancer. After labeling analytes with Girard's reagent T for adding positive charge, 145 N‐glycans compositions were identified by CE‐MS [133].

## 6 CONCLUSIONS

CE‐MS is bringing a promise for the analyses of complex samples such as urine. In some respects, it can be considered as the potential alternative to the commonly used HPLC‐MS and GC‐MS techniques. Different mechanism of separation allows targeting a different group of analytes. Besides high separation efficiency for the charged analytes, it offers low consumption of organic solvents and samples, low operating costs, short time analyses, and specific modes for the sample stacking of low abundant analytes.

The research reviewed above shows the potential of this technique to determine the drugs from urine, in several cases at a very low concentration level, determination of drug metabolites, drug enantiomers, and screening methods for the determination of drugs of abuse.

The specific application of CE‐MS includes metabolomic studies, providing data for the correlation between specific peptide and protein compositions in urine for several diseases (including diseases of the excretory tracts, cardiovascular system, bowel diseases, and many types of cancer). These recently published studies show CE‐MS as a suitable technique for biomarker discovery and show the possible use of CE‐MS as a diagnostic tool. The articles also discover the potential of CE‐MS to be used in clinical practice and complement the commonly used techniques of HPLC‐MS and GC‐MS.

## CONFLICT OF INTEREST

The authors have declared no conflict of interest.

## Data Availability

Data sharing is not applicable to this article as no new data were created or analyzed in this study.

## References

[1] Khamis MM , Adamko DJ , El‐Aneed A . Mass spectrometric based approaches in urine metabolomics and biomarker discovery. Mass Spectrom Rev. 2017;36:115–34. 25881008 10.1002/mas.21455

[2] García A , Godzien J , López‐Gonzálvez Á , Barbas C . Capillary electrophoresis mass spectrometry as a tool for untargeted metabolomics. Bioanalysis 2017;9:99–130. 27921456 10.4155/bio-2016-0216

[3] Wittke S , Fliser D , Haubitz M , Bartel S , Krebs R , Hausadel F , Hillmann M , Golovko I , Koester P , Haller H , Kaiser T , Mischak H , Weissinger EM . Determination of peptides and proteins in human urine with capillary electrophoresis–mass spectrometry, a suitable tool for the establishment of new diagnostic markers. J Chromatogr A. 2003;1013:173–81. 14604118 10.1016/s0021-9673(03)00713-1

[4] Ullsten S , Danielsson R , Bäckström D , Sjöberg P , Bergquist J . Urine profiling using capillary electrophoresis‐mass spectrometry and multivariate data analysis. J Chromatogr A. 2006;1117:87–93. 16620839 10.1016/j.chroma.2006.03.048

[5] Bouatra S , Aziat F , Mandal R , Guo AC , Wilson MR , Knox C , Bjorndahl TC , Krishnamurthy R , Saleem F , Liu P , Dame ZT , Poelzer J , Huynh J , Yallou FS , Psychogios N , Dong E , Bogumil R , Roehring C , Wishart DS . The human urine metabolome. PLoS One. 2013;8:e73076. 24023812 10.1371/journal.pone.0073076 PMC3762851

[6] Decramer S , de Peredo AG , Breuil B , Mischak H , Monsarrat B , Bascands J‐L , Schanstra JP . Urine in clinical proteomics. Mol Cell Proteomics. 2008;7:1850–62. 18667409 10.1074/mcp.R800001-MCP200

[7] Julian BA , Suzuki H , Suzuki Y , Tomino Y , Spasovski G , Novak J . Sources of urinary proteins and their analysis by urinary proteomics for the detection of biomarkers of disease. Proteomics Clin Appl. 2009;3:1029–43. 20161589 10.1002/prca.200800243 PMC2808139

[8] Saude EJ , Sykes BD . Urine stability for metabolomic studies: effects of preparation and storage. Metabolomics 2007;3:19–27.

[9] Kalantari S , Jafari A , Moradpoor R , Ghasemi E , Khalkhal E . Human urine proteomics: analytical techniques and clinical applications in renal diseases. Int J Proteomics. 2015;2015:1–17. 10.1155/2015/782798 PMC4677025 26693351

[10] Lenz EM , Bright J , Wilson ID , Hughes A , Morrisson J , Lindberg H , Lockton A . Metabonomics, dietary influences and cultural differences: a 1H NMR‐based study of urine samples obtained from healthy British and Swedish subjects. J Pharm Biomed Anal. 2004;36:841–9. 15533678 10.1016/j.jpba.2004.08.002

[11] Ferreira L , Sánchez‐Juanes F , González‐Ávila M , Cembrero‐Fuciños D , Herrero‐Hernández A , González‐Buitrago JM , Muñoz‐Bellido JL . Direct identification of urinary tract pathogens from urine samples by matrix‐assisted laser desorption ionization‐time of flight mass spectrometry. J Clin Microbiol. 2010;48:2110–5. 20392910 10.1128/JCM.02215-09 PMC2884468

[12] Premasiri WR , Clarke RH , Womble ME . Urine analysis by laser Raman spectroscopy. Lasers Surg Med. 2001;28:330–4. 11344513 10.1002/lsm.1058

[13] Shaw RA , Kotowich S , Mantsch HH , Leroux M . Quantitation of protein, creatinine, and urea in urine by near‐infrared spectroscopy. Clin Biochem. 1996;29:11–19. 8929818 10.1016/0009-9120(95)02011-x

[14] Yilmaz B , Arslan S . Determination of atenolol in human urine by using HPLC. Sep Sci PLUS. 2018;1:4–10.

[15] Woźniak MK , Wiergowski M , Aszyk J , Kubica P , Namieśnik J , Biziuk M . Application of gas chromatography–tandem mass spectrometry for the determination of amphetamine‐type stimulants in blood and urine. J Pharm Biomed Anal. 2018;148:58–64. 28957720 10.1016/j.jpba.2017.09.020

[16] Ryan D , Robards K , Prenzler PD , Kendall M . Recent and potential developments in the analysis of urine: a review. Anal Chim Acta. 2011;684:17–29. 10.1016/j.aca.2010.10.035 21167980

[17] Rose C , Parker A , Jefferson B , Cartmell E . The Characterization of feces and urine: a review of the literature to inform advanced treatment technology. Crit Rev Environ Sci Technol. 2015;45:1827–79. 26246784 10.1080/10643389.2014.1000761 PMC4500995

[18] NASA Technical Reports Server (NTRS) . [cited 2020 Dec 12]. Available from: https://ntrs.nasa.gov/citations/19710023044

[19] Kanbara A , Miura Y , Hyogo H , Chayama K , Seyama I . Effect of urine pH changed by dietary intervention on uric acid clearance mechanism of pH‐dependent excretion of urinary uric acid. Nutr J. 2012;11:39. 22676161 10.1186/1475-2891-11-39 PMC3406944

[20] Ure A. & A dictionary of chemistry and mineralogy: with their applications. T. Tegg & Son; London, 1831.

[21] Liu L , Mo H , Wei S , Raftery D . Quantitative analysis of urea in human urine and serum by 1H nuclear magnetic resonance. Analyst 2012;137:595–600. 22179722 10.1039/c2an15780b PMC4758351

[22] Shahbaz H , Gupta M . StatPearls. Treasure Island, FL: StatPearls Publishing ; 2020.

[23] Pundir CS , Kumar P , Jaiwal R . Biosensing methods for determination of creatinine: a review. Biosens Bioelectron. 2019;126: 707–24. 30551062 10.1016/j.bios.2018.11.031

[24] Kodani E , Inoue H , Atarashi H , Tomita H , Okumura K , Yamashita T , Origasa H . Predictive ability of creatinine clearance versus estimated glomerular filtration rate for outcomes in patients with non‐valvular atrial fibrillation: subanalysis of the J‐RHYTHM Registry. IJC Heart Vasc. 2020;29:100559. 10.1016/j.ijcha.2020.100559 PMC7298529 32566722

[25] Wagner BD , Accurso FJ , Laguna TA . The applicability of urinary creatinine as a method of specimen normalization in the cystic fibrosis population. J Cyst Fibros. 2010;9:212–6. 20227353 10.1016/j.jcf.2010.02.004 PMC2883692

[26] Beasley‐Green A . Urine proteomics in the era of mass spectrometry. Int Neurourol J. 2016;20:S70–5. 27915473 10.5213/inj.1612720.360 PMC5169090

[27] Zhao M , Li M , Yang Y , Guo Z , Sun Y , Shao C , Li M , Sun W , Gao Y . A comprehensive analysis and annotation of human normal urinary proteome. Sci Rep. 2017;7:3024. 28596590 10.1038/s41598-017-03226-6 PMC5465101

[28] Sarigul N , Korkmaz F , Kurultak İ . A new artificial urine protocol to better imitate human urine. Sci Rep. 2019;9:1–11. 31882896 10.1038/s41598-019-56693-4 PMC6934465

[29] Werth MT , Halouska S , Shortridge MD , Zhang B , Powers R . Analysis of metabolomic PCA data using tree diagrams. Anal Biochem. 2010;399:58–63. 20026297 10.1016/j.ab.2009.12.022 PMC2824058

[30] Mahadevan S , Shah SL , Marrie TJ , Slupsky CM . Analysis of metabolomic data using support vector machines. Anal Chem. 2008;80:7562–70. 18767870 10.1021/ac800954c

[31] Cook NR . Methods for evaluating novel biomarkers—a new paradigm. Int J Clin Pract. 2010;64:1723–7. 21070520 10.1111/j.1742-1241.2010.02469.x PMC3057673

[32] Gas B . In: Worsfold P , Townshend A , Poole C , editors. Electrophoresis Principles. Encyclopedia of analytical science (2nd ed.). Oxford: Elsevier; 2005. pp. 363–370.

[33] Foret F , Krivankova L , Bocek P . Capillary zone electrophoresis. Weinheim; New York: VCH Publishing; 1993.

[34] Niessen WMA , Tjaden UR , van der Greef J . Capillary electrophoresis—mass spectrometry. J Chromatogr A. 1993;636:3–19.

[35] Olivares JA , Nguyen NT , Yonker CR , Smith RD . On‐line mass spectrometric detection for capillary zone electrophoresis. Anal Chem. 1987;59:1230–2.

[36] Holtkamp H , Grabmann G , Hartinger CG . Electrophoretic separation techniques and their hyphenation to mass spectrometry in biological inorganic chemistry. Electrophoresis 2016;37:959–72. 26643265 10.1002/elps.201500502

[37] Foret F , Preisler J . Liquid phase interfacing and miniaturization in matrix‐assisted laser desorption/ionization mass spectrometry. Proteomics 2002;2:360–72. 12164695 10.1002/1615-9861(200204)2:4<360::AID-PROT360>3.0.CO;2-Y

[38] Hommerson P , Khan AM , de Jong GJ , Somsen GW . Ionization techniques in capillary electrophoresis‐mass spectrometry: principles, design, and application. Mass Spectrom Rev. 2011;30:1096–120. 21462232 10.1002/mas.20313

[39] Banks JF . Recent advances in capillary electrophoresis/electrospray/mass spectrometry. Electrophoresis 1997;18:2255–66. 9456040 10.1002/elps.1150181216

[40] Smith RD , Barinaga CJ , Udseth HR . Improved electrospray ionization interface for capillary zone electrophoresis‐mass spectrometry. Anal Chem. 1988;60:1948–52.

[41] Lee ED , Mück W , Henion JD , Covey TR . Liquid junction coupling for capillary zone electrophoresis/ion spray mass spectrometry. Biomed Environ Mass Spectrom. 1989;18:844–50.

[42] Krenkova J , Kleparnik K , Luksch J , Foret F . Microfabricated liquid junction hybrid capillary electrophoresis‐mass spectrometry interface for fully automated operation. Electrophoresis 2019;40:2263–70. 30794321 10.1002/elps.201900049

[43] Klepárník K , Otevřel M . Analyte transport in liquid junction nano‐electrospray interface between capillary electrophoresis and mass spectrometry. Electrophoresis 2010;31:879–85. 20191549 10.1002/elps.200900544

[44] Issaq HJ , Janini GM , Chan KC , Veenstra TD . Sheathless electrospray ionization interfaces for capillary electrophoresis–mass spectrometric detection: advantages and limitations. J Chromatogr A. 2004;1053:37–42. 15543970

[45] Wahl JH , Gale DC , Smith RD . Sheathless capillary electrophoresis‐electrospray ionization mass spectrometry using 10 μm I.D. capillaries: analyses of tryptic digests of cytochrome c. J Chromatogr A. 1994;659:217–22. 8118559 10.1016/0021-9673(94)85026-7

[46] Maxwell EJ , Chen DDY . Twenty years of interface development for capillary electrophoresis–electrospray ionization–mass spectrometry. Anal Chim Acta. 2008;627:25–33. 18790125 10.1016/j.aca.2008.06.034

[47] Janini GM , Conrads TP , Wilkens KL , Issaq HJ , Veenstra TD . A sheathless nanoflow electrospray interface for on‐line capillary electrophoresis mass spectrometry. Anal Chem. 2003;75:1615–9. 12705593 10.1021/ac020661+

[48] Tycova A , Vido M , Kovarikova P , Foret F . Interface‐free capillary electrophoresis‐mass spectrometry system with nanospray ionization—analysis of dexrazoxane in blood plasma. J Chromatogr A. 2016;1466:173–9. 27613146 10.1016/j.chroma.2016.08.042

[49] Řemínek R , Foret F , Chung DS . Application of capillary electrophoresis‐nano‐electrospray ionization‐mass spectrometry for the determination of N‐nitrosodimethylamine in pharmaceuticals. Electrophoresis 2021;42:334–41. 33368407 10.1002/elps.202000303

[50] Heemskerk AAM , Deelder AM , Mayboroda OA . CE–ESI‐MS for bottom‐up proteomics: advances in separation, interfacing and applications. Mass Spectrom Rev. 2016;35:259–71. 24852088 10.1002/mas.21432

[51] Liu CC , Zhang J , Dovichi NJ . A sheath‐flow nanospray interface for capillary electrophoresis/mass spectrometry. Rapid Commun Mass Spectrom. 2005;19:187–92. 15593250 10.1002/rcm.1769

[52] Wojcik R , Dada OO , Sadilek M , Dovichi NJ . Simplified capillary electrophoresis nanospray sheath‐flow interface for high efficiency and sensitive peptide analysis. Rapid Commun Mass Spectrom. 2010;24:2554–60. 20740530 10.1002/rcm.4672

[53] Liu J‐X , Zhang Y‐W , Yuan F , Chen H‐X , Zhang X‐X . Differential detection of Rhizoma coptidis by capillary electrophoresis electrospray ionization mass spectrometry with a nanospray interface. Electrophoresis 2014;35:3258–63. 25143257 10.1002/elps.201400334

[54] Ramautar R , Somsen GW , de Jong GJ . CE‐MS for metabolomics: developments and applications in the period 2016–2018. Electrophoresis 2019;40:165–79. 30232802 10.1002/elps.201800323 PMC6586046

[55] van Mever M , Hankemeier T , Ramautar R . CE–MS for anionic metabolic profiling: an overview of methodological developments. Electrophoresis 2019;40:2349–59. 31106868 10.1002/elps.201900115 PMC6771621

[56] Týčová A , Ledvina V , Klepárník K . Recent advances in CE‐MS coupling: instrumentation, methodology, and applications. Electrophoresis 2017;38:115–34. 27783411 10.1002/elps.201600366

[57] Zhang W, Ramautar R. CE‐MS for metabolomics: developments and applications in the period 2018–2020. *Electrophoresis* 2020;42. Available from: https://analyticalsciencejournals.onlinelibrary.wiley.com/doi/full/10.1002/elps.202000203. 10.1002/elps.202000203 PMC7891659 32906195

[58] Coon JJ , Zürbig P , Dakna M , Dominiczak AF , Decramer S , Fliser D , Frommberger M , Golovko I , Good DM , Herget‐Rosenthal S , Jankowski J , Julian BA , Kellmann M , Kolch W , Massy Z , Novak J , Rossing K , Schanstra JP , Schiffer E , Theodorescu D , Vanholder R , Weissinger EM , Mischak H , Schmitt‐Kopplin P . CE‐MS analysis of the human urinary proteome for biomarker discovery and disease diagnostics. PROTEOMICS – Clin Appl. 2008;2:964–73. 20130789 10.1002/prca.200800024 PMC2815342

[59] Julian BA , Wittke S , Novak J , Good DM , Coon JJ , Kellmann M , Zürbig P , Schiffer E , Haubitz M , Moldoveanu Z , Calcatera SM , Wyatt RJ , Sýkora J , Sládková E , Hes O , Mischak H , McGuire BM . Electrophoretic methods for analysis of urinary polypeptides in IgA‐associated renal diseases. Electrophoresis 2007;28:4469–83. 18004714 10.1002/elps.200700237

[60] Fernández‐Peralbo MA , Luque de Castro MD . Preparation of urine samples prior to targeted or untargeted metabolomics mass‐spectrometry analysis. TrAC Trends Anal Chem. 2012;41:75–85.

[61] Susa ST , Preuss CV . Drug metabolism. Treasure Island, FL: StatPearls Publishing; 2020. 28723052

[62] Hernández‐Mesa M , Cruces‐Blanco C , García‐Campaña AM . Capillary electrophoresis‐tandem mass spectrometry combined with molecularly imprinted solid phase extraction as useful tool for the monitoring of 5‐nitroimidazoles and their metabolites in urine samples. Talanta 2017;163:111–20. 27886759 10.1016/j.talanta.2016.10.092

[63] Lecoeur M , Rabenirina G , Schifano N , Odou P , Ethgen S , Lebuffe G , Foulon C . Determination of acetaminophen and its main metabolites in urine by capillary electrophoresis hyphenated to mass spectrometry. Talanta 2019;205:120108. 31450387 10.1016/j.talanta.2019.07.003

[64] Khan H , Gallant RC , Zamzam A , Jain S , Afxentiou S , Syed M , Kroezen Z , Shanmuganathan M , Britz‐McKibbin P , Rand ML , Ni H , Al‐Omran M , Qadura M . Personalization of aspirin therapy ex vivo in patients with atherosclerosis using light transmission aggregometry. Diagnostics 2020;10:871. 10.3390/diagnostics10110871 PMC7693608 33114560

[65] Šebestová A , Baron D , Pechancová R , Pluháček T , Petr J . Determination of oxaliplatin enantiomers at attomolar levels by capillary electrophoresis connected with inductively coupled plasma mass spectrometry. Talanta 2019;205:120151. 31450399 10.1016/j.talanta.2019.120151

[66] Piešťanský J , Maráková K , Galba J , Kováč A , Mikuš P . Comparison of hydrodynamically closed two‐dimensional capillary electrophoresis coupled with ultraviolet detection and hydrodynamically open capillary electrophoresis hyphenated with mass spectrometry in the bioanalysis of varenicline. J Sep Sci. 2017;40:2292–303. 28322496 10.1002/jssc.201700098

[67] Camperi J , De Cock B , Pichon V , Combes A , Guibourdenche J , Fournier T , Vander Heyden Y , Mangelings D , Delaunay N . First characterizations by capillary electrophoresis of human chorionic gonadotropin at the intact level. Talanta 2019;193:77–86. 30368301 10.1016/j.talanta.2018.09.095

[68] Maráková K , Piešťanský J , Zelinková Z , Mikuš P . Capillary electrophoresis hyphenated with mass spectrometry for determination of inflammatory bowel disease drugs in clinical urine samples. Molecules 2017;22:1973. 10.3390/molecules22111973 PMC6150202 29140288

[69] Shanmuganathan M , Macklai S , Cárdenas CB , Kroezen Z , Kim M , Zizek W , Lee H , Britz‐McKibbin P . High‐throughput and comprehensive drug surveillance using multisegment injection‐capillary electrophoresis‐mass spectrometry. JoVE J Vis Exp. 2019;146:e58986. 10.3791/58986 31081805

[70] DiBattista A , Rampersaud D , Lee H , Kim M , Britz‐McKibbin P . High throughput screening method for systematic surveillance of drugs of abuse by multisegment injection–capillary electrophoresis–mass spectrometry. Anal Chem. 2017;89:11853–61. 28981253 10.1021/acs.analchem.7b03590

[71] Pérez‐Alcaraz A , Borrull F , Aguilar C , Calull M , Benavente F . Enantiodetermination of R,S‐3,4‐methylenedioxypyrovalerone in urine samples by high pressure in‐line solid‐phase extraction capillary electrophoresis‐mass spectrometry. Talanta 2021;225:121994. 33592741 10.1016/j.talanta.2020.121994

[72] Ramautar R , Somsen GW , de Jong GJ . CE‐MS in metabolomics. Electrophoresis 2009;30:276–91. 19107702 10.1002/elps.200800512

[73] Ishibashi Y , Harada S , Takeuchi A , Iida M , Kurihara A , Kato S , Kuwabara K , Hirata A , Shibuki T , Okamura T , Sugiyama D , Sato A , Amano K , Hirayama A , Sugimoto M , Soga T , Tomita M , Takebayashi T . Reliability of urinary charged metabolite concentrations in a large‐scale cohort study using capillary electrophoresis‐mass spectrometry. Sci Rep. 2021;11:7407. 33795760 10.1038/s41598-021-86600-9 PMC8016858

[74] Saito R , Sugimoto M , Hirayama A , Soga T , Tomita M , Takebayashi T . Quality assessment of untargeted analytical data in a large‐scale metabolomic study. J Clin Med. 2021;10:1826. 33922230 10.3390/jcm10091826 PMC8122759

[75] Drouin N , van Mever M , Zhang W , Tobolkina E , Ferre S , Servais A‐C , Gou M‐J , Nyssen L , Fillet M , Lageveen‐Kammeijer GSM , Nouta J , Chetwynd AJ , Lynch I , Thorn JA , Meixner J , Lößner C , Taverna M , Liu S , Tran NT , Francois Y , Lechner A , Nehmé R , Al Hamoui Dit Banni G , Nasreddine R , Colas C , Lindner HH , Faserl K , Neusüß C , Nelke M , Lämmerer S , Perrin C , Bich‐Muracciole C , Barbas C , Gonzálvez ÁL , Guttman A , Szigeti M , Britz‐McKibbin P , Kroezen Z , Shanmuganathan M , Nemes P , Portero EP , Hankemeier T , Codesido S , González‐Ruiz V , Rudaz S , Ramautar R . Capillary electrophoresis‐mass spectrometry at trial by metabo‐ring: effective electrophoretic mobility for reproducible and robust compound annotation. Anal Chem. 2020;92:14103–12. 32961048 10.1021/acs.analchem.0c03129 PMC7581015

[76] Mamani‐Huanca M , de la Fuente AG , Otero A , Gradillas A , Godzien J , Barbas C , López‐Gonzálvez Á . Enhancing confidence of metabolite annotation in capillary electrophoresis‐mass spectrometry untargeted metabolomics with relative migration time and in‐source fragmentation. J Chromatogr A. 2021;635:461758. 10.1016/j.chroma.2020.461758 33302137

[77] Wild J , Shanmuganathan M , Hayashi M , Potter M , Britz‐McKibbin P . Metabolomics for improved treatment monitoring of phenylketonuria: urinary biomarkers for non‐invasive assessment of dietary adherence and nutritional deficiencies. Analyst 2019;144,:6595–608. 31608347 10.1039/c9an01642b

[78] Sasaki C , Hiraishi T , Oku T , Okuma K , Suzumura K , Hashimoto M , Ito H , Aramori I , Hirayama Y . Metabolomic approach to the exploration of biomarkers associated with disease activity in rheumatoid arthritis. PLoS One. 2019;14:e0219400. 31295280 10.1371/journal.pone.0219400 PMC6622493

[79] Pejchinovski M , Siwy J , Mullen W , Mischak H , Petri MA , Burkly LC , Wei R . Urine peptidomic biomarkers for diagnosis of patients with systematic lupus erythematosus. Lupus 2018;27:6–16. 28474961 10.1177/0961203317707827 PMC6037307

[80] Tailliar M , Schanstra JP , Dierckx T , Breuil B , Hanouna G , Charles N , Bascands J‐L , Dussol B , Vazi A , Chiche L , Siwy J , Faguer S , Daniel L , Daugas E , Jourde‐Chiche N . Urinary peptides as potential non‐invasive biomarkers for lupus nephritis: results of the peptidu‐LUP study. J Clin Med. 2021;10:1690. 33920017 10.3390/jcm10081690 PMC8071029

[81] Carleo A , Chorostowska‐Wynimko J , Koeck T , Mischak H , Czajkowska‐Malinowska M , Rozy A , Welte T , Janciauskiene S . Does urinary peptide content differ between COPD patients with and without inherited alpha‐1 antitrypsin deficiency? Int J Chron Obstruct Pulmon Dis. 2017;12:829–37. 28331304 10.2147/COPD.S125240 PMC5352160

[82] Weissinger EM , Human C , Metzger J , Hambach L , Wolf D , Greinix HT , Dickinson AM , Mullen W , Jonigk D , Kuzmina Z , Kreipe H , Schweier P , Böhm O , Türüchanow I , Ihlenburg‐Schwarz D , Raad J , Durban A , Schiemann M , Könecke C , Diedrich H , Holler E , Beutel G , Krauter J , Ganser A , Stadler M . The proteome pattern cGvHD_MS14 allows early and accurate prediction of chronic GvHD after allogeneic stem cell transplantation. Leukemia 2017;31:654–62. 27677743 10.1038/leu.2016.259

[83] Bannaga AS , Metzger J , Kyrou I , Voigtländer T , Book T , Melgarejo J , Latosinska A , Pejchinovski M , Staessen JA , Mischak H , Manns MP , Arasaradnam RP . Discovery, validation and sequencing of urinary peptides for diagnosis of liver fibrosis—a multicentre study. EBioMedicine. 2020;62:103083. 33160210 10.1016/j.ebiom.2020.103083 PMC7648178

[84] Omar M , Windhagen H , Krettek C , Ettinger M . Noninvasive diagnostic of periprosthetic joint infection by urinary peptide markers: a preliminary study. J Orthop Res. 2021;39:339–47. 33179279 10.1002/jor.24913

[85] Nkuipou‐Kenfack E , Schanstra JP , Bajwa S , Pejchinovski M , Vinel C , Dray C , Valet P , Bascands J‐L , Vlahou A , Koeck T , Borries M , Busch H , Bechtel‐Walz W , Huber TB , Rudolph KL , Pich A , Mischak H , Zürbig P . The use of urinary proteomics in the assessment of suitability of mouse models for ageing. PLoS One. 2017;12:e0166875. 28199320 10.1371/journal.pone.0166875 PMC5310860

[86] Zhang ZY , Nkuipou‐Kenfack E , Yang WY , Mujaj B , Thijs L , Latosinska A , Acloque E , Mischak H , Mebazaa A . P3506A novel urinary biomarker predicts 1 year mortality after discharge from Intensive Care. Eur Heart J. 2019;40. 10.1093/eurheartj/ehz745.0370. PMC6953276 31918764

[87] Gill B , Jobst K , Britz‐McKibbin P . Rapid screening of urinary 1‐hydroxypyrene glucuronide by multisegment injection–capillary electrophoresis–tandem mass spectrometry: a high‐throughput method for biomonitoring of recent smoke exposures. Anal Chem. 2020;92:13558–64. 32901481 10.1021/acs.analchem.0c03212

[88] Pontillo C , Mischak H . Urinary peptide‐based classifier CKD273: towards clinical application in chronic kidney disease. Clin Kidney J. 2017;10:192–201. 28694965 10.1093/ckj/sfx002 PMC5499684

[89] Levey AS , Coresh J . Chronic kidney disease. The Lancet. 2012;379:165–80. 10.1016/S0140-6736(11)60178-5 21840587

[90] Good DM , Zürbig P , Argilés À , Bauer HW , Behrens G , Coon JJ , Dakna M , Decramer S , Delles C , Dominiczak AF , Ehrich JHH , Eitner F , Fliser D , Frommberger M , Ganser A , Girolami MA , Golovko I , Gwinner W , Haubitz M , Herget‐Rosenthal S , Jankowski J , Jahn H , Jerums G , Julian BA , Kellmann M , Kliem V , Kolch W , Krolewski AS , Luppi M , Massy Z , Melter M , Neusüss C , Novak J , Peter K , Rossing K , Rupprecht H , Schanstra JP , Schiffer E , Stolzenburg J‐U , Tarnow L , Theodorescu D , Thongboonkerd V , Vanholder R , Weissinger EM , Mischak H , Schmitt‐Kopplin P . Naturally occurring human urinary peptides for use in diagnosis of chronic kidney disease. Mol Cell Proteomics. 2010;9:2424–37. 20616184 10.1074/mcp.M110.001917 PMC2984241

[91] Lindhardt M , Persson F , Oxlund C , Jacobsen IA , Zürbig P , Mischak H , Rossing P , Heerspink HJL . Predicting albuminuria response to spironolactone treatment with urinary proteomics in patients with type 2 diabetes and hypertension. Nephrol Dial Transplant Off Publ Eur Dial Transpl Assoc ‐ Eur Ren Assoc. 2018;33:296–303. 10.1093/ndt/gfw406 28064163

[92] Currie GE , von Scholten BJ , Mary S , Flores Guerrero J‐L , Lindhardt M , Reinhard H , Jacobsen PK , Mullen W , Parving H‐H , Mischak H , Rossing P , Delles C . Urinary proteomics for prediction of mortality in patients with type 2 diabetes and microalbuminuria. Cardiovasc Diabetol. 2018;17:50. 29625564 10.1186/s12933-018-0697-9 PMC5889591

[93] Magalhães P , Pejchinovski M , Markoska K , Banasik M , Klinger M , Švec‐Billá D , Rychlík I , Rroji M , Restivo A , Capasso G , Bob F , Schiller A , Ortiz A , Perez‐Gomez MV , Cannata P , Sanchez‐Niño MD , Naumovic R , Brkovic V , Polenakovic M , Mullen W , Vlahou A , Zürbig P , Pape L , Ferrario F , Denis C , Spasovski G , Mischak H , Schanstra JP . Association of kidney fibrosis with urinary peptides: a path towards non‐invasive liquid biopsies? Sci Rep. 2017;7:16915. 29208969 10.1038/s41598-017-17083-w PMC5717105

[94] Rodríguez‐Ortiz ME , Pontillo C , Rodríguez M , Zürbig P , Mischak H , Ortiz A . Novel urinary biomarkers for improved prediction of progressive eGFR loss in early chronic kidney disease stages and in high risk individuals without chronic kidney disease. Sci Rep. 2018;8:15940. 30374033 10.1038/s41598-018-34386-8 PMC6206033

[95] He T , Pejchinovski M , Mullen W , Beige J , Mischak H , Jankowski V . Peptides in plasma, urine, and dialysate: toward unravelling renal peptide handling. PROTEOMICS – Clin Appl. 2021;15:2000029. 10.1002/prca.202000029 32618437

[96] Magalhães P , Pontillo C , Pejchinovski M , Siwy J , Krochmal M , Makridakis M , Carrick E , Klein J , Mullen W , Jankowski J , Vlahou A , Mischak H , Schanstra JP , Zürbig P , Pape L . Comparison of urine and plasma peptidome indicates selectivity in renal peptide handling. PROTEOMICS ‐ Clin Appl. 2018;12:1700163. 10.1002/prca.201700163 29611317

[97] Fédou C , Breuil B , Golovko I , Decramer S , Magalhães P , Muller F , Dreux S , Zürbig P , Klein J , Schanstra JP , Buffin‐Meyer B . Comparison of the amniotic fluid and fetal urine peptidome for biomarker discovery in renal developmental disease. Sci Rep. 2020;10:21706. 33303833 10.1038/s41598-020-78730-3 PMC7729974

[98] Markoska K , Pejchinovski M , Pontillo C , Zürbig P , Jacobs L , Smith A , Masin‐Spasovska J , Stojceva‐Taneva O , Polenakovic M , Magni F , Mischak H , Spasovski G . Urinary peptide biomarker panel associated with an improvement in estimated glomerular filtration rate in chronic kidney disease patients. Nephrol Dial Transplant. 2018;33:751–9. 28992073 10.1093/ndt/gfx263

[99] Pejchinovski M , Siwy J , Metzger J , Dakna M , Mischak H , Klein J , Jankowski V , Bae KT , Chapman AB , Kistler AD . Urine peptidome analysis predicts risk of end‐stage renal disease and reveals proteolytic pathways involved in autosomal dominant polycystic kidney disease progression. Nephrol Dial Transplant. 2017;32:487–97. 27382111 10.1093/ndt/gfw243

[100] Kanzelmeyer NK , Zürbig P , Mischak H , Metzger J , Fichtner A , Ruszai KH , Seemann T , Hansen M , Wygoda S , Krupka K , Tönshoff B , Melk A , Pape L . Urinary proteomics to diagnose chronic active antibody‐mediated rejection in pediatric kidney transplantation—a pilot study. Transpl Int. 2019;32:28–37. 30357927 10.1111/tri.13363

[101] Siwy J , Zürbig P , Argiles A , Beige J , Haubitz M , Jankowski J , Julian BA , Linde PG , Marx D , Mischak H , Mullen W , Novak J , Ortiz A , Persson F , Pontillo C , Rossing P , Rupprecht H , Schanstra JP , Vlahou A , Vanholder R . Noninvasive diagnosis of chronic kidney diseases using urinary proteome analysis. Nephrol Dial Transplant. 2017;32: 2079–89. 27984204 10.1093/ndt/gfw337 PMC5837301

[102] Ricci P , Magalhães P , Krochmal M , Pejchinovski M , Daina E , Caruso MR , Goea L , Belczacka I , Remuzzi G , Umbhauer M , Drube J , Pape L , Mischak H , Decramer S , Schaefer F , Schanstra JP , Cereghini S , Zürbig P . Urinary proteome signature of renal cysts and diabetes syndrome in children. Sci Rep. 2019;9:2225. 30778115 10.1038/s41598-019-38713-5 PMC6379363

[103] Huang Z‐A , Scotland KB , Li Y , Tan J , Kung SHY , Chew BH , Chen DDY , Lange D . Determination of urinary prostaglandin E2 as a potential biomarker of ureteral stent associated inflammation. J Chromatogr B Analyt Technol Biomed Life Sci. 2020;1145:122107. 10.1016/j.jchromb.2020.122107 32315976

[104] Han X , Sanderson P , Nesheiwat S , Lin L , Yu Y , Zhang F , Amster IJ , Linhardt RJ . Structural analysis of urinary glycosaminoglycans from healthy human subjects. Glycobiology 2020;30:143–51. 31616929 10.1093/glycob/cwz088 PMC7415306

[105] Mishima E , Fukuda S , Mukawa C , Yuri A , Kanemitsu Y , Matsumoto Y , Akiyama Y , Fukuda NN , Tsukamoto H , Asaji K , Shima H , Kikuchi K , Suzuki C , Suzuki T , Tomioka Y , Soga T , Ito S , Abe T . Evaluation of the impact of gut microbiota on uremic solute accumulation by a CE‐TOFMS‐based metabolomics approach. Kidney Int. 2017;92: 634–45. 28396122 10.1016/j.kint.2017.02.011

[106] Pelander L , Brunchault V , Buffin‐Meyer B , Klein J , Breuil B , Zürbig P , Magalhães P , Mullen W , Elliott J , Syme H , Schanstra JP , Häggström J , Ljungvall I . Urinary peptidome analyses for the diagnosis of chronic kidney disease in dogs. Vet J. 2019;249: 73–79. 31239169 10.1016/j.tvjl.2019.05.010

[107] Baumgart DC , Carding SR . Inflammatory bowel disease: cause and immunobiology. Lancet. 2007;369:1627–40. 17499605 10.1016/S0140-6736(07)60750-8

[108] Maráková K , Piešťanský J , Zelinková Z , Mikuš P . Simultaneous determination of twelve biogenic amines in human urine as potential biomarkers of inflammatory bowel diseases by capillary electrophoresis‐tandem mass spectrometry. J Pharm Biomed Anal. 2020;186:113294. 32348953 10.1016/j.jpba.2020.113294

[109] Neurath MF . Cytokines in inflammatory bowel disease. Nat Rev Immunol. 2014;14:329–42. 24751956 10.1038/nri3661

[110] Yamamoto M , Shanmuganathan M , Hart L , Pai N , Britz‐McKibbin P . Urinary metabolites enable differential diagnosis and therapeutic monitoring of pediatric inflammatory bowel disease. Metabolites 2021;11:245. 33921143 10.3390/metabo11040245 PMC8071482

[111] Piestansky J , Matuskova M , Cizmarova I , Majerova P , Kovac A , Mikus P . Ultrasensitive determination of serotonin in human urine by a two dimensional capillary isotachophoresis‐capillary zone electrophoresis hyphenated with tandem mass spectrometry. J Chromatogr A. 2021;1648:462190. 33979756 10.1016/j.chroma.2021.462190

[112] Piestansky J , Olesova D , Galba J , Marakova K , Parrak V , Secnik P , Secnik P , Kovacech B , Kovac A , Zelinkova Z , Mikus P . Profiling of amino acids in urine samples of patients suffering from inflammatory bowel disease by capillary electrophoresis‐mass spectrometry. Mol Basel Switz. 2019;24:3345. 10.3390/molecules24183345. PMC6767150 31540027

[113] Oedit A , Hankemeier T , Lindenburg PW . On‐line coupling of two‐phase microelectroextraction to capillary electrophoresis–mass spectrometry for metabolomics analyses. Microchem J. 2021;162:105741.

[114] Yamamoto M , Pinto‐Sanchez MI , Bercik P , Britz‐McKibbin P . Metabolomics reveals elevated urinary excretion of collagen degradation and epithelial cell turnover products in irritable bowel syndrome patients. Metabolomics 2019;15:82. 31111238 10.1007/s11306-019-1543-0

[115] Gaziano T , Reddy KS , Paccaud F , Horton S , Chaturvedi V . Cardiovascular disease. The International Bank for Reconstruction and Development/The World Bank 2006. 21250342

[116] Campbell RT , Jasilek A , Mischak H , Nkuipou‐Kenfack E , Latosinska A , Welsh PI , Jackson CE , Cannon J , McConnachie A , Delles C , McMurray JJV . The novel urinary proteomic classifier HF1 has similar diagnostic and prognostic utility to BNP in heart failure. ESC Heart Fail. 2020;7:1595–604. 32383555 10.1002/ehf2.12708 PMC7373887

[117] Zhang Z‐Y , Nkuipou‐Kenfack E , Yang W‐Y , Wei F‐F , Cauwenberghs N , Thijs L , Huang Q‐F , Feng Y‐M , Schanstra JP , Kuznetsova T , Voigt J‐U , Verhamme P , Mischak H , Staessen JA . Epidemiologic observations guiding clinical application of a urinary peptidomic marker of diastolic left ventricular dysfunction. J Am Soc Hypertens. 2018;12:438–47. e4. 29681522 10.1016/j.jash.2018.03.007 PMC5990703

[118] Huang Q‐F , Keer JV , Zhang Z‐Y , Trenson S , Nkuipou‐Kenfack E , Aelst LNLV , Yang W‐Y , Thijs L , Wei F‐F , Ciarka A , Vanhaecke J , Janssens S , Cleemput JV , Mischak H , Staessen JA . Urinary proteomic signatures associated with β‐blockade and heart rate in heart transplant recipients. PLoS One. 2018;13:e0204439. 30248148 10.1371/journal.pone.0204439 PMC6152976

[119] He T , Siwy J , Metzger J , Mullen W , Mischak H , Schanstra JP , Zürbig P , Jankowski V . Associations of urinary polymeric immunoglobulin receptor peptides in the context of cardio‐renal syndrome. Sci Rep. 2020;10:8291. 32427855 10.1038/s41598-020-65154-2 PMC7237418

[120] Cieslarova Z , Magaldi M , Barros LA , do Lago CL , Oliveira DR , Fonseca FAH , Izar MC , Lopes AS , Tavares MFM , Klassen A . Capillary electrophoresis with dual diode array detection and tandem mass spectrometry to access cardiovascular biomarkers candidates in human urine: trimethylamine‐N‐oxide and L‐carnitine. J Chromatogr A. 2019;1583:136–42. 30509618 10.1016/j.chroma.2018.10.005

[121] Sheon M , Yvonne SH , Justyna S , William M , Ashok G . Christian D. Polymerization‐incompetent uromodulin in the pregnant stroke‐prone spontaneously hypertensive rat. Hypertension 2017;69:910–8. 28348009 10.1161/HYPERTENSIONAHA.116.08826 PMC5389592

[122] Ferley J , Soerjomataram I , Dikshit R , Eser S , Mathers C , Rebelo M , Parkin DM , Forman D , Bray F . Cancer incidence and mortality worldwide: Sources, methods and major patterns in GLOBOCAN 2012. Int J Cancer. 2015;136:E359–86. 25220842 10.1002/ijc.29210

[123] Belczacka I , Latosinska A , Siwy J , Metzger J , Merseburger AS , Mischak H , Vlahou A , Frantzi M , Jankowski V . Urinary CE‐MS peptide marker pattern for detection of solid tumors. Sci Rep. 2018;8:5227. 29588543 10.1038/s41598-018-23585-y PMC5869723

[124] Frantzi M , van Kessel KE , Zwarthoff EC , Marquez M , Rava M , Malats N , Merseburger AS , Katafigiotis I , Stravodimos K , Mullen W , Zoidakis J , Makridakis M , Pejchinovski M , Critselis E , Lichtinghagen R , Brand K , Dakna M , Roubelakis MG , Theodorescu D , Vlahou A , Mischak H , Anagnou NP . Development and validation of urine‐based peptide biomarker panels for detecting bladder cancer in a multi‐center study. Clin Cancer Res. 2016;22:4077–86. 27026199 10.1158/1078-0432.CCR-15-2715

[125] Theodorescu D , Schiffer E , Bauer HW , Douwes F , Eichhorn F , Polley R , Schmidt T , Schöfer W , Zürbig P , Good DM , Coon JJ , Mischak H . Discovery and validation of urinary biomarkers for prostate cancer. PROTEOMICS – Clin Appl. 2008;2:556–70. 19759844 10.1002/prca.200780082 PMC2744126

[126] Voigtländer T , Metzger J , Schönemeier B , Jäger M , Mischak H , Manns MP , Lankisch TO . A combined bile and urine proteomic test for cholangiocarcinoma diagnosis in patients with biliary strictures of unknown origin. United Eur. Gastroenterol J. 2017;5:668–76, 10.1177/2050640616687836. PMC5548357 28815030

[127] Voigtländer T , Metzger J , Husi H , Kirstein MM , Pejchinovski M , Latosinska A , Frantzi M , Mullen W , Book T , Mischak H , Manns MP . Bile and urine peptide marker profiles: access keys to molecular pathways and biological processes in cholangiocarcinoma. J Biomed Sci. 2020;27:13. 31900160 10.1186/s12929-019-0599-5 PMC6941325

[128] Udo R , Katsumata K , Kuwabara H , Enomoto M , Ishizaki T , Sunamura M , Nagakawa Y , Soya R , Sugimoto M , Tsuchida A . Urinary charged metabolite profiling of colorectal cancer using capillary electrophoresis‐mass spectrometry. Sci Rep. 2020;10:21057. 33273632 10.1038/s41598-020-78038-2 PMC7713069

[129] Frantzi M , Gomez EG , Pedregosa AB , Rosa JV , Latosinska A , Culig Z , Merseburger AS , Luque RM , Requena Tapia MJ , Mischak H , Carrasco Valiente J . CE–MS‐based urinary biomarkers to distinguish non‐significant from significant prostate cancer. Br J Cancer. 2019;120:1120–8. 31092909 10.1038/s41416-019-0472-z PMC6738044

[130] Moran AB , Domínguez‐Vega E , Nouta J , Pongracz T , de Reijke TM , Wuhrer M , Lageveen‐Kammeijer GSM . Profiling the proteoforms of urinary prostate‐specific antigen by capillary electrophoresis – mass spectrometry. J Proteomics. 2021;238:104148. 33618028 10.1016/j.jprot.2021.104148

[131] Kammeijer GSM , Nouta J , de la Rosette JJMCH , de Reijke TM , Wuhrer M . An in‐depth glycosylation assay for urinary prostate‐specific antigen. Anal Chem. 2018;90:4414–21. 29502397 10.1021/acs.analchem.7b04281 PMC5885261

[132] MacLennan MS , Kok MGM , Soliman L , So A , Hurtado‐Coll A , Chen DDY . Capillary electrophoresis‐mass spectrometry for targeted and untargeted analysis of the sub‐5 kDa urine metabolome of patients with prostate or bladder cancer: a feasibility study. J Chromatogr B. 2018;1074–1075:79–85. 10.1016/j.jchromb.2018.01.007 29334632

[133] Song W , Zhou X , Benktander JD , Gaunitz S , Zou G , Wang Z , Novotny MV , Jacobson SC . In‐depth compositional and structural characterization of n‐glycans derived from human urinary exosomes. Anal Chem. 2019;91:13528–37. 31539226 10.1021/acs.analchem.9b02620 PMC6834888

